# The proximal experience of gratitude

**DOI:** 10.1371/journal.pone.0179123

**Published:** 2017-07-07

**Authors:** Kristin Layous, Kate Sweeny, Christina Armenta, Soojung Na, Incheol Choi, Sonja Lyubomirsky

**Affiliations:** 1 Department of Psychology, California State University, East Bay, Hayward, California, United States of America; 2 Department of Psychology, University of California, Riverside, Riverside, California, United States of America; 3 Department of Psychology, Seoul National University, Seoul, South Korea; University of Melbourne, AUSTRALIA

## Abstract

Although a great deal of research has tested the longitudinal effects of regularly practicing gratitude, much less attention has been paid to the emotional landscape directly following engagement in gratitude exercises. In three studies, we explored the array of discrete emotions people experience after being prompted to express or recall gratitude. In Studies 1 and 2, two different gratitude exercises produced not only greater feelings of gratitude relative to two positive emotion control conditions (i.e., recalling relief), but also higher levels of other socially relevant states like elevation, connectedness, and indebtedness. In a third study, conducted in both the U.S. and S. Korea, we compared a gratitude exercise to another positive emotion elicitation (i.e., recalling a kind act) and to a neutral task, and again found that the gratitude exercise prompted greater gratitude, elevation, indebtedness, and guilt, but no more embarrassment or shame, than the two comparison conditions. Additionally, in all three studies, emodiversity and cluster analyses revealed that gratitude exercises led to the simultaneous experience of both pleasant and unpleasant socially-relevant states. In sum, although it may seem obvious that gratitude exercises would evoke grateful, positive states, a meta-analysis of our three studies revealed that gratitude exercises actually elicit a mixed emotional experience—one that simultaneously leads individuals to feel uplifted and indebted.

## Introduction

The importance of gratitude is emphasized in most religious, philosophical, and cultural traditions [1]. During the past two decades, psychological theory and empirical evidence have supported the longstanding and widespread contention that gratitude is a virtue worth cultivating (e.g., [2–4]). As testimony to the explosion of gratitude research in the psychology literature, at the time of writing, a search for “gratitude” as a keyword on PsycINFO yielded hundreds of peer-reviewed articles and books on the topic since 2003, and dozens of those are articles that either review or test the effects of gratitude exercises. However, despite the preponderance of evidence supporting the long-term benefits of gratitude for people’s happiness, health, and relationships [5], the psychological state immediately following a gratitude exercise has been left relatively unexplored. That is, do gratitude exercises uniquely or primarily invoke feelings of gratitude, or might they instead prompt an array of socially relevant thoughts and emotions—both pleasant and unpleasant—that ultimately produce gratitude’s beneficial effects?

### Gratitude and gratitude exercises

Gratitude has been defined as a general tendency for people to appreciate the good things in their lives (i.e., as a trait, habit, moral virtue, or coping resource; [2, 6]), as well as a transient emotion elicited from particular situations or reflections (i.e., felt gratitude [2]). Some researchers reserve the term gratitude only for those instances in which people recognize that they have received a benefit and attribute that benefit to another person (i.e., benefit-triggered gratitude [7]), whereas others include in their definition of gratitude instances of benefits received from non-person entities (e.g., God or nature [2]) or prefer the umbrella term “appreciation” to represent grateful feelings that do not necessarily have a source (e.g., being thankful for a good night’s rest [8]; see also [9]). For practical purposes, we allow that gratitude interventions or exercises can be directed toward any of these targets, but the current studies focus on benefit-triggered gratitude.

Although multiple variations of gratitude exercises appear in the literature, most promote grateful thinking or expression by asking people to either recount the “blessings” or good things in their lives (e.g., [2, 10, 11]) or write a letter of gratitude to someone who has helped them (e.g., 12). The first type—“counting blessings”—can either be practiced internally, communicated in a group or individual therapy session or to a close other, or listed in a gratitude journal. The second type—the gratitude letter—can be delivered to the target (e.g., in a “gratitude visit” [11]), but is often kept private to unconfound gratitude expression from the act of interpersonal sharing [12, 13]. The gratitude intervention’s frequency (once per week, once per day), duration (just once, over several months), and format (self-administered, therapist-administered) has varied across studies, as have the outcomes explored. Two types of gratitude exercises—each administered just once rather than repeatedly—were used in the current studies. In Study 1, participants wrote gratitude letters, and in Studies 1–3, participants wrote about a time in which they were grateful to someone, but did not actually address the note, to remain comparable to other conditions.

### Why care about gratitude exercises?

Trait gratitude is correlated with desirable outcomes such as higher relationship satisfaction [14], happiness [4], and prosociality [4]; fewer symptoms of physical illness [15]; and less envy and materialism [4]. Thus, in the past decade or so, researchers began to test whether experimentally prompting people to engage in gratitude exercises could lead to the positive outcomes enjoyed by those high in trait gratitude. Multiple experiments have demonstrated the benefits of expressing or recalling gratitude, including enhanced relationship quality [16–17], higher well-being ([2, 10, 18–22]; but see [23–25]; see [26] for a meta-analytic review]), and better physical health [2]. However, despite the growing evidence for the benefits of gratitude exercises (e.g., [5]), less is known about the process by which such exercises produce these positive outcomes. A first step in understanding why and how gratitude exercises promote positive and lasting change is to identify how the individual feels immediately after expressing or recalling gratitude.

### What do gratitude exercises induce?

Surprisingly little research has examined the proximal emotional experience of the person engaging in the gratitude exercise. The most obvious prediction is that gratitude exercises will induce feelings of gratitude, and indeed one study did find increases in gratitude immediately following a counting blessings task versus a neutral task [27]. Unexpectedly, this is the only study we found that reported a measure of gratitude immediately following a single gratitude exercise (but see [28–30] for gratitude measured after experimental induction tasks like receiving a favor from a confederate).

Another assumption is that gratitude exercises increase positive affect and decrease negative affect. Again, studies documenting the immediate effects of gratitude exercises on positive and negative affect are scarce. Only one experiment to our knowledge captured positive affect and negative affect directly after a single gratitude exercise, and this study found increases in positive affect, but nonsignificant decreases in negative affect [31]. In sum, due to the surprisingly limited number of studies that assess emotions directly following a gratitude exercise, the immediate experience of the person engaging in the gratitude exercise is largely unknown.

In the longer term, some studies have found that following weeks of practicing daily or weekly gratitude, people show increases in gratitude (as assessed by a composite of multiple gratitude measurements over time) relative to a neutral control group (e.g., [2, 32, 33]), although one study using this approach found no difference between groups in feelings of gratitude ([22]; see [26] for a meta-analysis). Similarly mixed evidence comes from investigations that have examined composites of positive and negative affect over time. After weeks of practicing gratitude, respondents sometimes show increases in positive affect (e.g., [2, 18, 34]), sometimes decreases in negative affect [2], and sometimes no change in either [24, 35].

Theory suggests that gratitude serves an important role in reinforcing and motivating positive behaviors [7]. For example, receiving a “thank you” rewards the performance of a kind act, promoting future kind acts. Likewise, recipients of the kind act may feel grateful, which could motivate them to pay back their benefactor or pay the kindness forward to a third party. Such uniquely social situations likely elicit a complex mix of socially relevant states—such as elevation, connectedness, indebtedness, or even guilt—that will motivate the person to engage in social behavior that restores the balance [36].

Consequently, given the inherently social nature of gratitude, perhaps gratitude exercises have inconsistent effects on emotions because they elicit a blend of discrete socially relevant emotions and states that are concealed when positive and negative emotions are aggregated [37]. For example, increases in a composite of positive emotions could obscure the fact that participants may have felt more grateful, but not any more joyful or excited [38]. Decreases in a composite of negative emotions may reflect that people felt less angry, worried, or upset but might have also felt more indebted and guilty. Similarly, composites that combine both positive and reverse-scored negative emotions could also mask the possibility that gratitude exercises elicit both pleasant (e.g., gratitude and connectedness) and unpleasant (e.g., indebtedness and guilt) states, as an individual may feel simultaneously pleased and uncomfortable after receiving an unexpected gift.

In sum, to understand the complex constellation of social emotions people experience after practicing gratitude, researchers need to capture the immediate cognitive and emotional response to a gratitude exercise and analyze discrete rather than aggregated emotions. In the current studies, we examine the effects of gratitude exercises (versus a positive emotion or neutral control activity) not only on gratitude but also on such specific states as elevation, connectedness to others, indebtedness, and guilt that are also likely linked to the grateful experience. Importantly, because gratitude is directed toward a specific target, not only do we expect gratitude exercises to elicit mixed emotions, but also that the emotions elicited will be social in nature. For example, although we do not expect gratitude exercises to elicit the emotion of frustration, we do expect them to elicit the unpleasant social emotions of indebtedness and guilt. Theoretical and empirical support for the role we expect these states to play in gratitude exercises is described below.

#### Elevation

Haidt (2003) argues that when people witness acts of moral excellence (e.g., a good deed that promotes the welfare of others), they feel elevated [39]. That is, they have a warm feeling in their chest and feel moved, uplifted, and optimistic about humanity. Similarly, they report a desire to be a better person and act prosocially, presumably to emulate the good deeds they witnessed. Although elevation is usually described as a response to witnessing non-self-relevant acts of virtue [40], we argue that thinking about times in which people have performed moral acts toward oneself can also prompt feelings of elevation. Specifically, we reason that because gratitude exercises prompt people to reflect on others’ good and generous acts, they may feel elevated in response to these exercises. To our knowledge, no study has explored elevation following a gratitude exercise.

#### Connectedness

Gratitude exercises might also stimulate people to feel more connected to others [32]. Indeed, trait gratitude has been found to reinforce new and existing relationships, strengthening the sense that one has meaningful connections to others (e.g., perceptions of social support [41]). Specifically, gratitude predicts more committed, longer lasting relationships [42], promotes relationship connection and satisfaction [14, 43] and leads to more relationship maintenance behaviors (e.g., spending time together [44]). Thus, we posit that gratitude exercises are perfectly positioned to promote feelings of connectedness to others—an emotionally-relevant state—as people reflect on what others have done for them.

#### Indebtedness

Sometimes receiving gifts of time, resources, or support from others makes people feel downright uncomfortable. Indeed, theories of social equity hold that people are motivated to maintain equity in their relationships, that inequity makes people feel uncomfortable, and that this discomfort motivates people to restore balance [45]. The distress experienced in response to inequity is a feeling of indebtedness [46]. Although trait levels of gratitude and indebtedness are inversely related [47, 48], state levels are often positively correlated [30, 49, 50] or not significantly related [51–53], but not inversely related. This evidence indicates that gratitude and indebtedness often co-occur, and that indebtedness may be a common response to gratitude exercises. Furthermore, different situations appear to promote varying levels of indebtedness versus gratitude [50, 53, 54]. In line with equity theory, we predict that gratitude exercises will stimulate feelings of indebtedness in response to the generosity of the target of gratitude. Indeed, Emmons and Crumpler (2000) state that “to be genuinely grateful is to feel indebted for a debt that can never be repaid” (p. 58 [55]).

#### Guilt

Like gratitude, guilt is considered a social emotion that can arise from an interpersonal transaction [56]. Although guilt is often associated with moral transgressions, similar to indebtedness, it also results from positive inequities [56]. Although indebtedness is correlated with guilt, indebtedness is also associated with positive emotions like gratitude and even happiness, whereas guilt is most associated with negative emotions like feeling flustered and uneasy [50, 53]. We expect that reflecting on a benefactor’s kindness may lead people to feel guilty for failing to reciprocate or for not thanking him or her sooner.

### The current studies

Three studies examined an array of emotional responses to a variety of gratitude exercises. We tested the following hypotheses:

(1) *Gratitude exercises will induce more socially-relevant pleasant and unpleasant emotions overall than will relief exercises*, *a kindness exercise*, *or a neutral exercise*. Specifically, we hypothesized that participants would report higher levels of gratitude, elevation, and connectedness—but also higher levels of indebtedness and guilt—after writing about gratitude than after writing about a time they were relieved (Studies 1 and 2), a time they were kind (Study 3), or what they did last week (a neutral comparison; Study 3). The one exception is that we did not predict that the gratitude and kindness conditions would differ in connectedness, because these two conditions are similarly social. Studies 1 and 2 used a control condition that was similar to the gratitude condition(s) in structure, valence, and even the experience of good fortune—namely, a relief condition. Importantly, reflecting on relief experiences is a particularly strong control to reflecting on gratitude experiences because both types of experiences elicit mixed emotions (i.e., relief occurs when one narrowly escapes a bad event, which could make one feel uncomfortable and ponder how things could have turned out differently [57]). However, relief is generally directed toward a benefit received from an abstract force, whereas gratitude is typically directed toward a benefit received from another person, thus, triggering social emotions.

Consequently, we predicted—and tested in the first two studies—that expressing or recalling gratitude will elicit a mixed response of socially-oriented emotions. Study 3 employed both a neutral control and a positive emotion control to ensure that group differences found in Studies 1 and 2 were not just a result of our unique relief comparison conditions. The recalling kindness condition used in Study 3 was also a strong comparison to recalling gratitude, because both gratitude and kindness similarly involve social interactions that could boost connectedness. Recalling a past act of kindness also has the potential to increase negative emotions, as participants wonder whether their kind act was well received or appreciated and what they could have done differently (cf. [58]). We also included the neutral writing task in Study 3 to test a comparison activity that we expected to evoke neither positive nor negative emotions. Finally, we included two different types of gratitude exercises (gratitude recollection and gratitude letter) in our studies to bolster the robustness and generalizability of our findings.

Thus, across our three studies, we were able to explore the effects of receiving a social versus nonsocial benefit (gratitude versus relief), engaging in a social interaction in which one receives versus gives a benefit (gratitude versus kindness), and simply recounting the events of one’s week (neutral). These comparison conditions help illuminate the immediate experience of gratitude by comparing specific core elements of gratitude to other exercises that include some, but not all of these core elements.

(2) *Gratitude exercises will induce more mixed socially-relevant emotions (i*.*e*., *simultaneously pleasant and unpleasant emotions) than will relief*, *kindness*, *or neutral exercises*. To test this hypothesis, we employed two techniques. First, we used a relatively new type of analysis to evaluate the variety and evenness of emotions—namely, their “emodiversity” [59, 60]. The emodiversity equation quantifies the number of specific emotions experienced and the degree to which these specific emotions are experienced in the same proportion. The equation and SPSS syntax we used is available at http://www.emodiversity.org. If both positive and negative emotions are included in the equation, an emodiversity score indicates the degree to which participants experienced even amounts of positive and negative emotions (i.e., mixed emotions).

Second, we conducted K-means cluster analyses on social emotions to explore whether participants in certain conditions were more likely to exhibit particular profiles of emotions (e.g., whether those who engage in gratitude exercises exhibit high levels of both pleasant [moved and uplifted] and unpleasant [indebted] social emotions).

## Study 1

### Method

#### Participants

Participants from Amazon’s Mechanical Turk (mTurk; *N* = 138, 45.7% female) completed the study. Participants were mostly Asian (50.4%) and White (29.8%), with fewer Black/African American (5.3%), American Indian/Alaskan Native (3.1%), Hispanic/Latino (2.3%) participants, and participants who chose “other” (3.8%) or more than one category (5.3%). Ages ranged from 18 to 72 (*M* = 31.56, *SD* = 11.15).

#### Measures and procedure

We obtained approval from the Institutional Review Board at the University of California, Riverside to conduct this study. Before collecting data, we conducted a power analysis using the “pwr” package in R to determine data-stopping rules [61]. We sought to collect data from 50 participants per cell, which would yield 80% power at a medium effect size. We also used these data stopping rules for Study 2. Participants accessed our online study through a posting on mTurk and were immediately prompted with a consent form. After granting consent, participants were randomly assigned to one of three writing conditions: *gratitude experience* (*n* = 53), *gratitude letter* (*n* = 34), or *near-miss relief experience* (*n* = 50).

Our unequal sample sizes in Studies 1 and 2 are likely due to chance and a technicality of how participants were assigned to condition. In Study 1, the three conditions did not differ by gender, χ^2^(2) = 1.54, *p* = .46, ethnicity, χ^2^(12) = 7.78, *p* = .80, or age, *F*(2, 128) = 0.09, *p* = .91. In Study 2, the three conditions also did not differ by ethnicity, χ^2^(8) = 6.02, *p* = .65, or age, *F*(2, 126) = 0.99, *p* = .37, but did differ by gender such that the task-completion relief condition had similar numbers of men and women (*n* = 17 and *n* = 18, respectively), but the near-miss relief (*n* = 35 and *n* = 16, respectively) and gratitude experience (*n* = 33 and *n* = 10, respectively) conditions included more men than women, χ^2^(2) = 7.10, *p* = .03.

In Study 1, seven or eight people were filtered out of each condition due to failure to complete the writing task (*n* = 22), and the filtering did not significantly differ by condition, χ^2^(2) = 0.43, *p* = .81, gender, χ^2^(1) = 0.86, *p* = .36, or ethnicity, χ^2^(6) = 10.17, *p* = .12, but did differ by age such that people who got filtered were younger (*M*_*age*_ = 26.64, *SD* = 5.55) than those who did not (*M*_*age*_ = 31.56, *SD* = 11.15), *t*(151) = -2.03, *p* = 0.05. The numbers of participants presented for each condition are the number of participants who were included in the analyses and participant statistics. The results are not changed when analyses are run on the unfiltered sample.

Participants in the gratitude experience condition were instructed to write about an experience in which “someone did something for you for which you were truly grateful.” Sample responses included participants’ partners helping them with chores or taking them on a special outing or teachers giving extra help in an academic course. Participants in the gratitude letter condition were also instructed to “think back over the past several years of your life and remember an instance when someone did something for you for which you are extremely grateful,” but instead of just recounting the experience, they were asked to write a letter to that person (but not deliver it). Lastly, participants in the relief condition were instructed to write about an experience in which they “narrowly avoided a bad outcome and felt relief as a result” [57]. Examples included thinking they had lost something but then finding it, narrowly avoiding a bike or car accident, or thinking they had sent a compromising email to the wrong person.

After writing about their experience of relief or gratitude, participants indicated how they felt “right now” on 7-point Likert-type scales (1 = *not at all*, 7 = *extremely*). Because the term “elevation” was unlikely to be widely familiar (i.e., it is not a prototypical emotion like joy or anger [62]), we measured elevation by asking about the emotions theoretically associated with elevation [39]—namely, *moved* and *uplifted* [63]. Participants also indicated how *grateful*, *relieved*, *connected to other people*, *indebted*, and *guilty* they felt. In all studies, *indebted* was defined for participants as “feeling like you need to repay another for their actions that benefitted you.” In addition, participants rated a variety of emotions that we did not expect to be differentially elicited by the gratitude and relief conditions (*happy*, *worried/anxious*, *angry*, *frustrated*, *depressed/blue*, *joyful*, *nervous*, *inspired*, *scared*, *upset*, *unhappy*, and *pleased*) in an effort to test our hypotheses with specificity. Finally, we formed separate composites with all of the above positive (*grateful*, *relieved*, *moved*, *uplifted*, *connected to others*, *happy*, *pleased*, *joyful*, *and inspired*; Cronbach’s α = 0.88) and negative (*indebted*, *guilty*, *frustrated*, *depressed/blue*, *worried/anxious*, *angry*, *nervous*, *scared*, *unhappy*, and *upset*; α = 0.89) emotions.

We also measured the thoughts, physiological response, and volitional response theoretically associated with elevation—that is, *optimistic about humanity*, *a warm feeling in your chest*, *a desire to become a better person*, *and a desire to help others* [63], but these items were not centrally important to our mixed emotions hypotheses. That said, when combined with *moved* and *uplifted* in an elevation composite, these items yield high reliability (Cronbach’s *α* = .86) and show all the same trends as do *moved* and *uplifted* (analyses on this elevation composite across all four studies are included in Table A in S1 File). Although feeling moved and uplifted are social but not exclusively social emotions [36], in these studies, they are serving as a proxy for elevation, which is a social emotion whose label is not readily understandable by most.

The measures from all three experiments, as well as complete instructions for all manipulations, are included in S1 File. In addition, we included some measures that are not reported in the current paper, either because they addressed hypotheses not relevant to the current studies or were unnecessary to include for the sake of brevity.

### Results and discussion

#### Between group comparisons on discrete emotions (Hypothesis 1)

Group means, standard deviations, and results from our planned contrast (gratitude experience = +1, gratitude letter = +1, near-miss relief experience = -2 [64]) and pairwise contrasts are reported in Table 1. In addition, we include additional pairwise contrasts not mentioned in the text for interested readers. We conducted omnibus one-way ANOVAs on variables we did not expect to differ by group (e.g., happy, worried). The 95% confidence intervals across studies were created around the *rs* by converting *rs* to *Zrs* and using the equation $Zr\pm \frac{1.96}{\surd (n-3)}$ and then converting back to *r* [65].

**Table 1:** Descriptive statistics and contrast tests for Study 1.

	Experimental Conditions								
	Gratitude Experience		Gratitude Letter		Near-Miss Relief		*Contrast 1*	*Contrast 2*	*Contrast 3*
	*Contrast 1*: *+1*		*Contrast 1*: *+1*		*Contrast 1*: *-2*				
	*Contrast 2*: *+1*		*Contrast 2*: *0*		*Contrast 2*: *-1*				
	*Contrast 3*: *0*		*Contrast 3*: *+1*		*Contrast 3*: *-1*				
	*M(SD)*	*n*	*M(SD)*	*n*	*M(SD)*	*n*	*t-*contrast or *F-*statistic	*t-*contrast	*t*-contrast
**Grateful**	5.68 (1.60)	53	5.97 (1.09)	34	5.38 (1.37)	50	1.84^†^	1.02	2.20*
**Indebted**	4.55 (1.96)	53	4.59 (1.94)	34	3.60 (1.73)	50	2.89**	2.57*	2.38*
**Guilty**	1.81 (1.42)	53	1.82 (1.34)	34	2.28 (1.51)	50	-1.80^†^	-1.66^†^	-1.43
**Moved**	4.23 (1.73)	53	4.56 (1.56)	34	3.58 (1.99)	50	2.53*	1.83^†^	2.46*
**Uplifted**	4.32 (1.70)	53	4.88 (1.65)	33	3.88 (1.78)	50	2.33*	1.30	2.59*
**Connected to others**	5.33 (1.47)	52	5.01 (1.51)	34	4.40 (1.67)	49	2.75**	3.00**	1.77^†^
**Relieved**	4.30 (2.06)	53	4.82 (1.69)	33	5.04 (1.71)	49	1.44	2.02*	0.53
Happy	5.49 (1.54)	53	5.74 (1.40)	34	5.41 (1.38)	49	0.53		
Worried/anxious	2.17 (1.67)	53	2.32 (1.77)	34	2.48 (1.50)	50	0.46		
Angry	1.51 (1.34)	53	1.59 (1.16)	34	1.90 (1.28)	50	1.30		
Frustrated	1.85 (1.57)	53	1.91 (1.42)	34	2.30 (1.57)	50	1.23		
Depressed/blue	1.85 (1.54)	53	1.91 (1.44)	34	2.22 (1.66)	50	0.80		
Joyful	4.58 (1.83)	53	5.21 (1.32)	34	4.64 (1.54)	50	1.75		
Nervous	1.83 (1.40)	53	1.91 (1.22)	34	2.14 (1.47)	49	0.68		
Inspired	4.40 (1.80)	53	5.24 (1.68)	33	4.18 (1.77)	50	3.84*		
Scared	1.94 (1.51)	53	2.06 (1.56)	34	2.20 (1.55)	50	0.36		
Upset	1.81 (1.48)	53	1.79 (1.39)	34	2.18 (1.51)	50	1.04		
Unhappy	1.91 (1.54)	53	1.74 (1.05)	34	2.22 (1.54)	50	1.27		
Pleased	4.72 (1.73)	53	4.91 (1.58)	34	4.62 (1.65)	50	0.31		
Positive emotion composite	4.78 (1.34)	53	5.14 (1.05)	34	4.57 (1.05)	50	2.38^†^		
Negative emotion composite	2.12 (1.05)	53	2.16 (0.97)	34	2.35 (1.18)	50	0.64		
Emodiversity: All emotions	2.39 (0.28)	52	2.43 (0.30)	31	2.45 (0.50)	46	0.34		
**Emodiversity: Social emotions**	1.56 (0.18)	51	1.55 (0.25)	33	1.43 (0.41)	49	1.85^†^	1.90^†^	1.58

*Note*: A *t-*contrast is provided for emotions on which we performed a focused test (bolded in the table). An omnibus *F*-statistic is reported for all other emotions. We expected the relief condition to be higher on “relieved,” so the contrast analyses are reversed for that variable. We found no significant differences between the two gratitude conditions on any of our hypothesized variables and therefore do not report those specific results. The degrees of freedom for the emodiversity are fewer than for the composites because the composites allow for missing items (we stipulated that at least 5 items needed to be present for the positive and negative composites), whereas the emodiversity equation does not tolerate any missingness.

^†^*p* ≤ .10.

*p ≤ .05.

**p ≤ .01.

***p ≤ .001.

Results from our planned contrasts indicated that participants in the gratitude conditions reported feeling marginally more grateful, *t*(100.58) = 1.84, *p* = .07, *r* = .18, 95% CI [-.01, .36], and significantly more moved, *t*(134) = 2.53, *p* = .01, *r* = .21, [.05, .37], uplifted, *t*(133) = 2.33, *p* = .02, *r* = .20, [.03, .36], connected to others, *t*(132) = 2.75, *p* = .007, *r* = .23, [.07, .39], and indebted, *t*(134) = 2.89, *p* = .005, *r* = .24, [.08, .39], than those in the relief condition. Illustrating how composites can mask effects on discrete emotions, we found marginal group differences on the positive emotion composite, *F*(2, 134) = 2.38, *p* = .10, and nonsignificant differences on the negative emotion composite, *F*(2, 134) = 0.64, *p* = .53.

Unexpectedly, and opposite to our prediction, participants in the relief condition reported feeling marginally more guilty than those in the gratitude conditions, *t*(134) = -1.80, *p* = .07, *r* = -.15, 95% CI [-.31, .01]. We originally hypothesized that those recalling a benefactor’s kind acts would feel more guilt because someone had gone out of their way to help them. Instead, people who reflected on near-miss relief experiences may have felt that they should have known better or should have anticipated the narrowly avoided adverse event, and therefore felt guilty as a result. Supporting this explanation, participants in the relief condition trended toward being angrier and more frustrated, depressed, and upset than those in the gratitude conditions. See Tables B, F, and L in S1 File for correlations among all measured variables in all studies.

As expected, the gratitude and relief conditions showed similar means on most other positive (i.e., happy, joyful, and pleased; *F*s < 1.75) and negative (i.e., worried/anxious, angry, frustrated, depressed/blue, scared, upset, and nervous; *F*s < 1.30) emotions, but differed with respect to feeling inspired, *F*(2, 133) = 3.84, *p* = .02. Interestingly, a contrast predicting that the relief condition (+2) would report feeling more relieved than the two gratitude conditions (-1), was not significant, *t*(132) = 1.44, *p* = .15, *r* = .12, [-.05, .28], possibly because many of the grateful experiences contained elements of relief (i.e., people received help when they needed it most, thereby eliciting relief). For example, if a coworker helped on a difficult work assignment, one might feel grateful to the person, but simultaneously relieved that the project is completed. Indeed, feelings of gratitude and relief were highly correlated across our sample (*r* = .52, *p* < .001).

#### Between-group comparisons on profiles of mixed emotions (Hypothesis 2)

Although the above analyses largely support our first hypothesis that gratitude leads to both positive and ostensibly negative social emotions (i.e., feelings of being moved, uplifted, connected, and grateful, but also indebted), our interpretation is predicated on analyzing mean levels of discrete positive and negative emotions across individuals rather than the degree to which any one participant feels simultaneously positive and negative. Thus, our results could also be explained by some people feeling extremely positive after expressing gratitude and others feeling extremely negative, with their combination sufficient to yield a condition effect on both positive and negative emotions. The next set of analyses illuminates whether individual participants feel positive and negative at the same time. We explored this question in two ways—via emodiversity analyses and via cluster analyses.

First, using Quoidbach et al.’s (2014) formula [59], we calculated two emodiversity variables: one including all positive and negative emotions and one including just social emotions (see bolded items in Table 1 for means by condition). Conceptually, because we expected gratitude and relief to elicit similarly mixed emotions overall, the conditions should have similar emodiversity when including all emotions. However, if gratitude elicits more intense social emotions, with greater spread across them, as we predict, gratitude conditions should have greater emodiversity than relief conditions when only including social emotions. As expected, the three conditions did not differ on the emodiversity of all emotions, *F*(2, 72.80) = 0.72, but differed marginally in the predicted manner on our focused test, *t*(65.31) = 1.85, *p* = .07, *r* = .22, 95% CI [-.02, .44].

Second, to explore whether the different conditions elicited varying patterns of emotions, we conducted K-means cluster analyses on the same social emotions included in our emodiversity analyses and derived a two-cluster solution. Although many plausible combinations of emotions exist, a visual inspection of a plot of the within-groups sum of squares by number of clusters extracted, as well as centroid plots of various cluster solutions, demonstrated that a two-cluster solution was the most interpretable. We used this approach to derive number of clusters throughout the remaining studies. The majority of the sample (Cluster 1; *n* = 85) exhibited above average levels of feeling grateful, indebted, guilty, moved, uplifted, and connected to others. The other cluster (Cluster 2; *n* = 49) felt below average levels of all of the social emotions except for guilt. Although the majority of all three conditions were in Cluster 1, a larger percentage of people from the gratitude experience (63.46%) and gratitude letter (78.79%) conditions were in Cluster 1 than from the near-miss relief condition (53.06%), yielding a marginally significant chi-square test, χ^2^(2) = 5.63, *p* = .06. See Tables C and D in S1 File for cluster centroids (i.e., the average of the points in the cluster for each emotion), means and standard deviations of each emotion across conditions (for comparison to centroids), and frequencies by condition and cluster.

## Study 2

The findings from Study 1 supported our hypothesis that a gratitude exercise (compared to a relief comparison group) would promote elevation, gratitude, connectedness, indebtedness, and socially-oriented emodiversity. Contrary to our hypothesis, the gratitude groups reported marginally less guilt than the relief control group. In Study 2, we sought to replicate the findings from Study 1. However, this time we included a second relief control condition to ensure that Study 1’s findings were not an artifact of the specific type of comparison condition we used. Specifically, the type of relief described in the Study 1 control prompt was closest to near-miss relief [57], and thus we also included a task-completion relief prompt in Study 2. The two types of relief experiences were not expected to produce differences in our variables of interest.

### Method

#### Participants

Participants from mTurk (*N* = 130, 34.1% female) completed our study. Participants were mostly Asian (45.7%) and White (45.0%), with smaller numbers of Black/African American (3.9%) and American Indian/Alaskan Native (2.3%) participants, and participants who identified as more than one ethnicity (3.1%). Ages ranged from 18 to 75 (*M* = 31.53, *SD* = 10.17).

#### Measures and procedure

We obtained approval from the Institutional Review Board at the University of California, Riverside to conduct this study. Participants again accessed our online study from a link in mTurk and were met with a consent form upon clicking on the link. After providing consent, they were randomly assigned to one of three writing tasks. Two groups mirrored our conditions from Study 1: *gratitude experience* (*n* = 44) and *near-miss relief experience* (*n* = 51). Participants in the *task-completion relief experience* condition (*n* = 35) were instructed to write about an experience in which they had “finished an unpleasant task and felt relief as a result.” Sample responses included passing an exam, completing a presentation, or completing a workout program. Participants completed the same measures as in Study 1 and we again formed separate composites with all of the positive (*grateful*, *relieved*, *moved*, *uplifted*, *connected to others*, *happy*, *pleased*, *joyful*, *and inspired*; Cronbach’s α = 0.90) and negative (*indebted*, *guilty*, *frustrated*, *depressed/blue*, *worried/anxious*, *angry*, *nervous*, *scared*, *unhappy*, and *upset*; α = 0.92) emotions.

In Study 2, we again filtered out people (19 total) who did not correctly complete the writing assignment. The filtering did not significantly differ by condition, χ^2^(2) = 4.23, *p* = .12. Filtering varied significantly by gender (*n* = 2 for women; *n* = 17 for men), χ^2^(1) = 4.30, *p* = .04. Filtering also varied significantly by ethnicity, such that Asians (*n* = 12) were more likely to get filtered than any other group, χ^2^(4) = 18.84, *p* = .001. Lastly, filtering differed by age, such that people who got filtered were younger (*M*_*age*_ = 25.26, *SD* = 6.53) than those who did not (*M*_*age*_ = 31.53, *SD* = 10.17), *t*(146) = -2.60, *p* = 0.01. The numbers of participants presented for each condition are the number of participants who were included in the analyses and participant statistics. The results are not changed when analyses are run on the unfiltered sample. All participants who began the study also completed it.

### Results and discussion

#### Between group comparisons on discrete emotions (Hypothesis 1)

Group means, standard deviations, and results from our planned contrast tests (gratitude experience = +2, near-miss relief experience = -1, task-completion relief experience = -1) are reported in Table 2. We again conducted omnibus one-way ANOVAs on variables we did not expect to differ by group.

**Table 2:** Descriptive statistics and contrast tests for Study 2.

	Experimental Conditions								
	Gratitude Experience		Near-Miss Relief		Task-Completion Relief		*Contrast 1*	*Contrast 2*	*Contrast 3*
	*Contrast 1*: *+2*		*Contrast 1*: *-1*		*Contrast 1*: *-1*				
	*Contrast 2*: *+1*		*Contrast 2*: *-1*		*Contrast 2*: *0*				
	*Contrast 3*: *+1*		*Contrast 3*: *0*		*Contrast 3*: *-1*				
	*M(SD)*	*n*	*M(SD)*	*n*	*M(SD)*	*n*	*t-*contrast or *F-*statistic	*t-*contrast	*t*-contrast
**Grateful**	5.70 (1.44)	44	4.92 (1.67)	51	5.09 (1.76)	35	2.32*	2.35*	1.69^†^
**Indebted**	4.84 (1.61)	44	3.22 (1.91)	51	3.03 (1.93)	32	5.02***	4.35***	4.29***
**Guilty**	1.77 (1.34)	43	2.14 (1.52)	51	1.94 (1.56)	34	-0.98	-1.21	-0.51
**Moved**	4.45 (1.73)	44	3.80 (1.94)	51	3.29 (1.84)	35	2.65**	1.71^†^	2.80**
**Uplifted**	4.70 (1.71)	44	4.02 (1.85)	51	4.06 (1.68)	35	2.03*	1.90^†^	1.63^†^
**Connected to others**	5.36 (1.29)	44	4.06 (1.97)	51	4.44 (1.94)	34	3.39***	3.61***	2.30*
**Relieved**	4.27 (1.76)	44	4.76 (2.06)	50	4.91 (1.62)	35	1.64^†^	1.28	1.54
Happy	5.32 (1.49)	44	4.92 (1.72)	51	5.03 (1.79)	35	0.70		
Worried/anxious	2.20 (1.36)	44	2.62 (1.70)	50	2.51 (1.87)	35	0.93		
Angry	1.70 (1.36)	44	2.04 (1.51)	50	1.91 (1.44)	34	0.64		
Frustrated	1.74 (1.26)	43	2.26 (1.72)	50	1.97 (1.36)	34	1.38		
Depressed/blue	1.80 (1.36)	44	2.24 (1.82)	51	2.23 (1.68)	35	1.22		
Joyful	4.86 (1.62)	44	3.94 (2.08)	51	4.03 (2.08)	35	3.56*		
Nervous	1.80 (1.34)	44	2.26 (1.59)	50	2.17 (1.77)	35	1.12		
Inspired	4.68 (1.83)	44	3.88 (2.05)	51	4.20 (1.89)	35	2.03		
Scared	1.70 (1.42)	44	2.20 (1.58)	51	2.06 (1.64)	35	1.24		
Upset	1.57 (1.27)	44	2.22 (1.73)	50	1.97 (1.58)	35	2.32		
Unhappy	1.73 (1.21)	44	1.92 (1.47)	51	1.66 (1.37)	35	0.45		
Pleased	4.75 (1.56)	44	4.20 (1.97)	51	4.53 (1.58)	34	1.16		
Positive emotion composite	4.90 (1.17)	44	4.28 (1.43)	51	4.39 (1.34)	35	2.86^†^		
Negative emotion composite	2.09 (1.08)	44	2.32 (1.32)	51	2.13 (1.30)	35	0.45		
Emodiversity: All emotions	2.44 (0.25)	42	2.32 (0.51)	45	2.39 (0.41)	29	0.90		
**Emodiversity: Social emotions**	1.56 (0.28)	43	1.40 (0.41)	51	1.37 (0.41)	30	2.76**	2.26*	2.20*

*Note*: A *t-*contrast is provided for emotions on which we performed a focused test (bolded in the table). An omnibus *F*-statistic is reported for all other emotions. We expected the relief condition to be higher on “relieved,” so the contrast analyses are reversed for that variable. We found no significant differences between the relief conditions on any of our hypothesized variables and therefore do not report those specific results. The degrees of freedom for the emodiversity are fewer than for the composites because the composites allow for missing items (we stipulated that at least 5 items needed to be present for the positive and negative composites), whereas the emodiversity equation does not tolerate any missingness.

^†^*p* ≤ .10.

*p ≤ .05.

**p ≤ .01.

***p ≤ .001.

Corroborating the results from Study 1, participants who wrote about a gratitude experience reported feeling significantly more grateful, *t*(127) = 2.32, *p* = .02, *r* = .20, 95% CI [.03, .36], moved, *t*(127) = 2.65, *p* = .009, *r* = .23, [.06, .39], uplifted, *t*(127) = 2.03, *p* = .04, *r* = .18, [.01, .34], connected to others, *t*(126) = 3.39, *p* = .001, *r* = .29, [.12, .44], and indebted, *t*(124) = 5.02, *p* < .001, *r* = .41, [.25, .55], than the two relief groups. We again found a marginal difference among conditions on the positive emotion composite, *F*(2, 126) = 2.86, *p* = .06, and a nonsignificant difference among conditions for the negative emotion composite, *F*(2, 79.41) = 0.44, *p* = .64.

Counter to our prediction and different from the finding in Study 1, participants in the gratitude condition did not differ in their reports of guilt from those in the relief conditions, *t*(125) = -.98, *p* = .33, *r* = -.09, 95% CI [-.26, .09]. Similar to Study 1, participants in the gratitude and relief conditions did not differ significantly on all other positive (*F*s < 2.03) and negative (*F*s < 2.32) emotions we measured, except for joyful, *F*(2, 78.68) = 3.56, *p* = .03. A contrast predicting that the two relief conditions (+1) elicited more relief than the gratitude condition (-2) was marginally significant, *t*(126) *=* 1.64, *p* = .10, *r* = .14, [-.03, .31].

#### Between-group comparisons on profiles of mixed emotions (Hypothesis 2)

As in our first study, we again tested whether the emodiversity of all emotions and just social emotions varied by condition (see bottom of Table 2 for means and standard deviations). Replicating our results from Study 1, we found no significant group differences on the emodiversity of all emotions, *F*(2, 62.10) = 0.90, *p* = .41, but did find our predicted group difference on the emodiversity of social emotions, *t*(102.66) = 2.76, *p* = .007, *r* = .26, 95% CI [.08, .43].

Also replicating Study 1, we again derived a two-factor solution in our K-means cluster analyses, with one cluster reporting above average levels of every emotion (except for guilt, which was right at the average; Cluster 1, *n* = 64) and another cluster with mostly below average levels of every emotion (except for guilt, which was again at the average; Cluster 2, *n* = 60). Like in Study 1, participants in the gratitude experience condition were more likely to be in Cluster 1 (62.79%) than Cluster 2, whereas those in the near-miss relief condition were equally likely to be in Cluster 1 (49.02%) and Cluster 2. Participants in the task-completion relief condition were less likely to be in Cluster 1 (40%) than Cluster 2. Although the observed proportions are in line with our prediction, there were no significant differences in cluster membership by condition, χ^2^(2) = 3.91, *p* = .14. See Tables G and H in S1 File for cluster centroids and frequencies by condition and cluster.

## Study 3

Our first two studies demonstrated that recalling a grateful experience evoked stronger patterns of socially relevant pleasant and unpleasant emotions than recalling another mixed emotional experience—a relief experience. In our third study, conducted in both the U.S. and S. Korea, we compared a gratitude exercise to an emotionally-neutral control activity and to another ostensibly positive emotion exercise—namely, writing about a kind act one has performed.

We believe that recalling one’s own kindness is a particularly strong comparison to recalling one’s gratitude because, unlike relief, kindness is a similarly social experience as gratitude, allowing us to explore whether it is just social connection that renders gratitude emotionally complex or whether it is something unique to gratitude itself (i.e., to receiving versus giving a benefit). Although recalling kindness may conceivably trigger negative thoughts (e.g., Did I too much (or too little)? Was my help unappreciated or misinterpreted? [58]), we expected that gratitude would be more likely to evoke our proposed blend of socially-oriented positive and negative emotions (e.g., feeling indebted, guilty, moved, and uplifted).

First, we predicted that the gratitude condition would lead participants to feel more grateful, moved, uplifted, indebted, guilty, embarrassed, and ashamed than would the kindness or control conditions. Second, we expected the gratitude condition to elicit more social emodiversity and more clusters of simultaneous pleasant and unpleasant social emotions than the kindness or control conditions. Given past research showing that repeated expressions of gratitude fail to increase well-being in S. Korea [13], we expected that mixed emotions in response to the gratitude activity would be particularly strong in our S. Korean sample (e.g., a condition by culture interaction). Lastly, because gratitude and kindness (unlike relief) are equally social, we expected these two conditions to elicit similar levels of connectedness; to this end, we ran an omnibus *F*-test to explore differences among conditions rather than the focused tests run in Studies 1 and 2.

### Method

#### Participants

Participants were students at the University of California, Riverside (UCR; *n* = 194) who obtained course credit in exchange for participation (70.5% Female) and students at Seoul National University (SNU; *n* = 233) in South Korea who received 5,000 won (just over $4.00) in exchange for participation (54.08% Female). In the U.S. sample, ages ranged from 18 to 44 (*M*_age_ = 19.35, *SD*_*age*_ = 2.28) and the sample was ethnically diverse with 43.3% Asian, 33.5% Hispanic/Latino, 8.2% identifying as “more than one” ethnicity, 7.2% White, 4.1% “Other”, 2.1% Black/African American, 0.5% Hawaiian/Pacific Islander, 0.5% American Indian/Alaskan Native, and 0.5% missing. In the S. Korean sample, ages ranged from 18 to 31 (*M*_age_ = 22.02, *SD*_*age*_ = 2.68) and ethnicity was not collected due to the homogenous student body. We increased our sample size in this study and were well-powered (99%) to detect medium effect sizes.

#### Measures and procedure

We obtained approval from the Institutional Review Boards at the University of California, Riverside and Seoul National University to conduct this study. Due to small effect sizes in Studies 1 and 2, we decided to increase our sample size in Study 3 to be more confident in our effects, so we sought to have 70 participants per condition for 90% power to detect medium effects. Participants obtained a link to our study via the Department of Psychology’s subject pool website at UCR and via a general online community at SNU. Upon accessing the study, participants provided consent, were randomly assigned to condition, and then completed one of three writing tasks for 8 minutes: recalling a time in which they were grateful (*gratitude* condition; *n* = 148), a time in which they were kind (*kindness* condition; *n* = 136), or what they did over the past seven days (*control* condition; *n* = 143; complete instructions are included in the S1 File).

In the U.S. sample, 207 participants initially logged on to take the study, with 13 yielding no or insufficient data. Those who dropped out before completing the study did not vary by condition, χ^2^(2) = 2.85, *p* = .24, which was randomly assigned upon entry to the website. In the S. Korean sample, 279 participants started the study, with 46 yielding no or insufficient data. Like the U.S. sample, those who dropped in the S. Korean sample did not significantly vary by condition, χ^2^(2) = 5.30, *p* = .07.

After completing the assigned writing activity, participants reported the degree to which they felt many of the same positive (i.e., *grateful*, *moved*, *uplifted*, *happy*, *pleased*, *joyful*, *and enjoyment/fun*; Cronbach’s α = 0.89) and negative (*indebted*, *guilty*, *embarrassed*, *ashamed*, *frustrated*, *depressed/blue*, *worried/anxious*, *angry*, and *unhappy*; α = 0.82) emotions as in the first two studies. We again also computed separate composites for positive and negative emotions, the emodiversity of all emotions listed above, and the emodiversity of just the social emotions (i.e., *grateful*, *moved*, *uplifted*, *indebted*, *guilty*, *embarrassed*, *ashamed*).

We also included a 6-item measure of state connectedness (adapted from the subscale of the Balanced Measure of Psychological Needs [66]; Cronbach’s α = .66) including statements like “I feel close and connected with other people who are important to me” (1 = *no agreement*, 5 = *much agreement*), and a 6-item measure of state gratitude (GQ-6 [4]; α = .86), including statements like “Right now I feel that I have much in life to be thankful for” (1 = *strongly disagree*, 7 = *strongly disagree*).

### Results and discussion

#### Between-group comparisons on discrete emotions (Hypothesis 1)

Group means, standard deviations, and results from our planned contrast analyses are reported in Table 3. As predicted, contrast analyses revealed that participants in the gratitude (+1) condition reported feeling more grateful, *t*(202.62) = 8.01, *p* < .001, *r* = .49, 95% CI [.38, .59], indebted, *t*(424) = 8.71, *p* < .001, *r* = .39, [.31, .47], guilty, *t*(281.54) = 2.21, *p* = .03, *r* = .13, [.01, .24], moved, *t*(267.42) = 6.38, *p* < .001, *r* = .36, [.26, .46], and uplifted, *t*(281.76) = 3.19, *p* = .002, *r* = .19, [.07, .30], than participants in the kindness (-1) condition. However, contrary to our predictions, contrast analyses revealed that participants in the gratitude condition did not report feeling significantly more embarrassed, *t*(424) = -1.44, *p* = .15, or ashamed, *t*(277.92) = -0.20, *p* = .84, than participants in the kindness condition. Surprisingly, the gratitude group also did not report higher levels of gratitude on our multi-item measure of gratitude (the GQ-6) than the kindness group, *t*(424) = -0.28, *p* = .78.

**Table 3:** Means (SDs) and contrast tests for Study 3.

	Experimental Conditions								
	Gratitude		Kindness		Control		*Contrast 1*	*Contrast 2*	*Contrast 3*
	*Contrast 1*: *+1*		*Contrast 1*: *-1*		*Contrast 1*: *0*				
	*Contrast 2*: *+1*		*Contrast 2*: *0*		*Contrast 2*: *-1*				
	*Contrast 3*: 0		*Contrast 3*: 1		*Contrast 3*: *-1*				
	*M(SD)*	*n*	*M(SD)*	*n*	*M(SD)*	*n*	*t-*contrast or *F-*statistic	*t-*contrast	*t*-contrast
**Grateful (**single-item)	6.36 (0.85)	148	5.14 (1.59)	136	4.35 (1.66)	142	8.01***	12.99***	4.09***
**Gratitude** (multi-item)	5.57 (1.00)	148	5.61 (1.03)	136	5.39 (1.05)	143	-0.28	1.54	1.79^†^
**Indebted**	4.61 (1.88)	148	2.79 (1.79)	136	2.43 (1.60)	143	8.71***	10.57***	1.71^†^
**Guilty**	2.61 (1.78)	148	2.17 (1.57)	136	2.63 (1.80)	142	2.21*	-0.12	-2.30*
**Moved**	5.57 (1.22)	148	4.57 (1.42)	136	2.97 (1.64)	143	6.38***	15.36***	8.73***
**Uplifted**	5.16 (1.52)	148	4.60 (1.44)	136	3.31 (1.62)	143	3.19**	10.00***	7.00***
**Embarrassed**	2.37 (1.34)	148	2.40 (1.39)	136	2.76 (1.62)	143	-1.44	-0.20	1.24
**Ashamed**	2.94 (1.58)	148	3.21 (1.50)	136	2.97 (1.65)	143	-0.20	-2.25*	-2.00*
Connectedness (multi-item)	3.85 (0.62)	148	3.73 (0.64)	136	3.70 (0.70)	143	2.06		
Happy	5.75 (1.30)	147	5.09 (1.55)	135	4.21 (1.57)	141	40.54***		
Worried/anxious	2.94 (1.71)	148	2.91 (1.70)	136	4.03 (1.70)	143	20.20***		
Angry	1.63 (1.11)	148	1.51 (0.99)	136	1.91 (1.26)	143	4.70**		
Frustrated	2.03 (1.52)	148	1.95 (1.36)	136	2.72 (1.72)	143	9.67**		
Depressed/blue	2.24 (1.43)	148	2.15 (1.51)	136	2.62 (1.72)	143	3.15*		
Joyful	4.16 (1.55)	148	4.06 (1.50)	136	3.55 (1.53)	143	6.69***		
Unhappy	1.95 (1.42)	148	1.95 (1.40)	136	2.30 (1.49)	143	2.82^†^		
Pleased	4.60 (1.53)	147	4.38 (1.40)	136	3.84 (1.45)	143	10.29***		
Enjoyment/fun	3.70 (1.71)	148	3.51 (1.54)	136	3.33 (1.51)	143	2.01		
Positive emotion composite	5.04 (1.00)	148	4.48 (1.16)	136	3.65 (1.19)	143	57.17***		
Negative emotion composite	2.59 (0.98)	148	2.34 (0.94)	136	2.71 (1.10)	143	4.90**		
Emodiversity: All emotions	2.36 (0.24)	146	2.32 (0.29)	135	2.33 (0.32)	140	0.95		
**Emodiversity: Social emotions**	1.65 (0.22)	148	1.58 (0.31)	136	1.45 (0.42)	141	2.22*	5.00***	2.91**

*Note*: A *t-*contrast is provided for emotions on which we performed a focused test (bolded in the table). An omnibus *F*-statistic is reported for all other emotions. The degrees of freedom for the emodiversity analyses are fewer than for the composites because the composites allow for some missing items (we allowed three [out of seven] missing item in the positive emotion composite and up to four [out of nine] missing items in the negative emotion composite), whereas the emodiversity equation does not tolerate any missingness.

^†^*p* ≤ .10.

*p ≤ .05.

**p ≤ .01.

***p ≤ .001.

Also as predicted, participants in the gratitude (+1) condition reported feeling more grateful, *t*(208.66) = 12.99, *p* < .001, *r* = .67, 95% CI [.59, .74], indebted, *t*(424) = 10.57, *p* < .001, *r* = .46, [.38, .53], moved, *t*(262.71) = 15.36, *p* < .001, *r* = .69, [.62, .75], and uplifted, *t*(286.37) = 10.00, *p* < .001, *r* = .51, [.42, .59], than participants in the control (-1) condition. Contrary to our predictions, participants in the gratitude condition did not report feeling more guilty, *t*(287.18) = -0.12, *p* = .90, *r* = .01, [-.11, .12], embarrassed, *t*(424) = -0.20, *p* = .85, *r* = .01, [-.09, .25], or ashamed, *t*(275.42) = -2.25, *p* = .03, *r* = -.13, [-.25, -.02] than those in the control condition.

As expected from previous research on positive activities, we found significant omnibus effects on participants feeling happy, joyful, and pleased, and on the positive emotion composite (all *F*s > 6.69), with means indicating that the gratitude and kindness groups manifested the highest levels of positive emotions, yet no significant group differences on the multi-item state connectedness measure or on feelings of enjoyment/fun, *F* < 2.06. Lastly, we found significant group differences on most non-social negative emotions (angry, depressed/blue, frustrated, and worry, and on the negative emotion composite), *F*s > 3.15, and a marginal effect on feeling unhappy, *F*(2, 424) = 2.82, *p* = .06, which were largely driven by the control group having higher levels of non-social negative emotions than the gratitude and kindness groups. Perhaps the gratitude and kindness conditions dampened feelings of frustration, worry, anger, depression and unhappiness, or perhaps recounting what one did over the course of the week (or what one did not accomplish) evoked such feelings.

#### Culture analyses

The condition effects we reported above remained intact when we explored the effects of culture (U.S. versus S. Korea) and its interaction with condition (gratitude, kindness, control) in factorial ANOVAs. We found significant condition by culture interactions on the items grateful, indebted, moved, uplifted, depressed/blue, and the positive emotion composite (*F*s > 3.23), as well as marginal interactions on guilty, worried/anxious, unhappy, and the negative emotion composite (*F*s > 2.54). The means revealed that the interactions were largely driven by the S. Koreans performing differently in the control group than their U.S. counterparts. Specifically, the S. Korean sample experienced the control task (i.e., writing about their last 7 days) as more positive, but also with more guilt, depression, worry, and unhappiness than the U.S. sample, possibly because their academic lives at SNU are widely recognized to be highly stressful. Additionally, counter to our prediction, the interaction involving indebtedness and the negative emotion composite appeared to be driven by the S. Korean sample feeling less indebted and less negative overall in the gratitude and kindness conditions than the U.S. sample, but about equally indebted and negative in the control group. Means by culture and condition are reported in Tables J and K in S1 File. We found no condition by culture interactions on people feeling embarrassed, ashamed, connected, happy, angry, frustrated, joyful, or pleased, or on their levels of enjoyment/fun (*F* < 2.05).

Additionally, we found a main effect of culture on feeling indebted, uplifted, embarrassed, ashamed, connected, joyful, and the negative emotion composite (*F* > 5.17), and a marginal main effect of culture on feeling moved, enjoyment/fun, and on the positive emotion composite (*F* > 2.77), such that S. Koreans overall felt more positive and less negative than their U.S. counterparts. We found no main effect of culture on feeling grateful, guilty, happy, worried/anxious, angry, frustrated, depressed/blue, unhappy, pleased (*F* < 2.49).

#### Between-group comparisons on profiles of mixed emotions (Hypothesis 2)

As in our other studies, we again wanted to explore the effect of the gratitude condition (versus the kindness and control conditions) on eliciting a profile of mixed emotions via emodiversity and cluster analyses. As expected, we found no group differences on the emodiversity of all emotions, *F*(2, 271.54) = 0.95, *p* = .39, but the gratitude group showed higher emodiversity of social emotions relative to the kindness group, *t*(243.96) = 2.22, *p* = .03, *r* = .14, 95% CI [.02, .26], and the control group, *t*(209.50) = 5.00, *p* < .001, *r* = .33 [.20, .44].

To explore different patterns of responding, we again conducted a K-means cluster analysis on the same positive and negative emotions included in our social emodiversity analyses (grateful, moved, uplifted, indebted, guilty, embarrassed, and ashamed). In contrast with the two-cluster solutions in the first two studies, we found that a three-cluster solution best represented the data. The majority of the sample (Cluster 1; *n* = 175) exhibited above average levels of feeling grateful, moved, and uplifted, but below average levels of feeling indebted, guilty, embarrassed, and ashamed. Thus, the first cluster felt more positive than negative. The next largest group (Cluster 2; *n* = 128) exhibited below average levels of most emotions, except about average levels of feeling embarrassed and ashamed. Thus, the second cluster was feeling about average, and not as positive as the first cluster. The last group (Cluster 3; *n* = 122) exhibited truly mixed emotions, with above average levels on both positive and negative emotions.

Cluster membership varied significantly by condition, χ^2^(4) = 123.70, *p* < .001. Importantly, people from the gratitude group were almost equally distributed across Cluster 1, feeling mostly positive (50.00%), and Cluster 3, feeling relatively high levels of both positive and negative emotions (44.59%). The gratitude group constituted over half of Cluster 3, with the rest of Cluster 3 being split about evenly across the kindness and control conditions. The majority of people in the control group were in Cluster 2 (62.41%), feeling less emotional than the rest of the sample, whereas only 5.41% of people from the gratitude group were in this group. Unsurprisingly, people in the kindness group were most likely to be in Cluster 1 (52.94%), with the rest being equally likely to be in Clusters 2 and 3. Thus, although gratitude and kindness elicitations were about equally likely to influence the Cluster 1 pattern of positivity, gratitude was twice as likely to evoke high levels of both positive and negative social emotions. See Tables O and P in S1 File for cluster centroids and frequencies by condition and cluster.

#### Culture analyses

Again, we conducted factorial ANOVAs to explore potential effects of culture and its interaction with condition. An analysis of the emodiversity of all emotions revealed a condition by culture interaction, such that S. Koreans were about equally emodiverse as their U.S. counterparts in the gratitude group, less emodiverse in the kindness groups, and more emodiverse in the control group, *F*(2, 415) = 4.20, *p* = .02. We found no evidence for a main effect of condition or culture on the emodiversity of all emotions, *F*(2, 415) = 0.89, *p* = .41 and *F*(1, 415) = 0.73, *p* = .39, respectively. For the emodiversity of social emotions, we again found our predicted main effect of condition, *F*(2, 419) = 14.77, *p* < .001, no main effect of culture, *F*(1, 419) = 2.33, *p* = .13, and a marginal condition by culture interaction, such that contrary to our prediction, South Koreans were less emodiverse than their U.S. counterparts in the gratitude and kindness groups, but more emodiverse in the control group, *F*(2, 419) = 2.76, *p* = .06. Means and standard deviations by condition and culture are included in Tables J and K in S1 File.

We also included cluster analyses by culture in Tables Q-T in S1 File. For the S. Korean sample, we again derived a three-cluster solution that mirrored the clusters in the combined sample. For the U.S. sample, we derived a two-cluster solution, with the first cluster representing an average of Cluster 1 and Cluster 3 from the combined sample (mixed emotions) and the second cluster representing Cluster 2 from the combined sample, with relatively lower levels of all emotions. The representation of condition in each cluster within each culture mirrored those presented in the combined sample.

### Meta-analysis of hypothesized emotions

To summarize our findings across our three studies, as well as to address potential statistical power concerns in Studies 1 and 2, we meta-analytically combined the effect sizes from our contrast analyses (see Table 4; see [65, 67] for procedures). For each study, we used the contrast analyses presented in the paper (listed as “Contrast 1” in Tables 1 through 3). To review, in Study 1, we compared two gratitude conditions (+1 each) to a relief condition (-2); in Study 2, we compared one gratitude condition (+2) to two relief conditions (-1 each); and in Study 3, we compared a gratitude condition (+1) to a kindness condition (-1), setting aside the control condition.

**Table 4:** Summary of meta-analytic findings.

	Sample Size		Weighted *r* effect size	Unweighted *r* effect size			*p*-value	
Variable	Number of Studies (k)	Total *N*	Mean [95% CI]	Mean [95% CI]	Min	Max	Fixed Effects Model	Random Effects
								Model
Grateful	3	439.2	.34 [.26, .42]	.30 [-.06, .59]	.18	.49	*Z* = 6.68, *p* < .001	*t*(2) = 2.69, *p* = .06
Indebted	3	688	.37 [.30, .43]	.35 [.17, .50]	.24	.41	*Z* = 9.22, *p* < .001	*t*(2) = 6.14, *p* = .01
Guilty	3	549.54	.01 [-.07, .09]	-.04 [-.30, .23]	-.15	.13	Z = 1.27, *p* = .10	*t*(2) = -0.43, *p* = .35
Moved	3	537.42	.29 [.22, .37]	.27 [.11, .42]	.21	.36	Z = 6.50, *p* < .001	*t*(2) = 5.31, *p* = .02
Uplifted	3	550.76	.19 [.10, .27]	.19 [.17, .21]	.18	.20	*Z* = 4.31, *p* < .001	*t*(2) = 30.48, *p* < .001
Connected to others	3	688	.15 [.07, .22]	.20 [-.08, .45]	.08	.29	*Z* = 4.36, *p* < .001	*t*(2) = 3.05, *p* = .05
Emodiversity: Social emotions	3	420.93	.18 [.09, .28]	.21 [.09, .32]	.14	.26	*Z* = 3.89, *p* < .001	*t*(2) = 5.67, *p* = .01

*Note*: We included only *Contrast 1* from each study (the contrast illustrated in the manuscript text and in the tables), as it best represented our hypotheses. Specifically, in Study 1, we compared the Gratitude Experience (+1) and Gratitude Letter (+1) conditions to the Near Miss Relief condition (-2). In Study 2, we compared the Gratitude Experience (+2) condition to the Near-Miss Relief (-1) and Task-Completion Relief (-1) conditions. Lastly, in Study 3, we compared the Gratitude condition (+1) to the Kindness (-1) condition (setting the control condition aside).

Total *N* per study is the *N* that was included in the statistical analyses. Due to heterogeneous variances on some of our variables, we calculated a *t* that did not assume homogeneity of variance and therefore our degrees of freedom were reduced for some variables. We calculated the weighted and unweighted *r*s by converting all *r*s to *Zrs* and using the following formulas: $\bar{Zr}=\frac{\sum Zr(n-3)}{\sum \left(n-3\right)}$ and $\bar{Zr}=\frac{\sum Zr}{k}$, respectively, in which *n* is the number of participants in each study and *k* is the number of studies. We calculated confidence intervals around the weighted and unweighted $\bar{Zr}$ with the following formulas: $\bar{Zr}\pm \frac{1.96}{\sqrt{\sum \left(n-3\right)}}$ and $\bar{Zr}\pm t_{critical}*\left(\frac{{SD}_{Zr}}{\sqrt{k}}\right)$, respectively. We then converted all *Zr*s to *r*s. The weighted $\bar{Zr}$ corresponds to a fixed effects approach and the unweighted $\bar{Zr}$ corresponds to a random effects approach. To calculate the fixed effects *p*-value, we found the *Z* that corresponded to the one-tailed *p*-value of each variable in each study and combined them with the following equation: $Z=\frac{\sum Z}{\surd k}$ (The Stouffer Method). If the *t-*statistic was opposite to our prediction, we attributed a *p*-value of .5 and *Z* value of zero. Our Z estimates for some studies are a bit conservative due to online *p*-value to *Z* calculators and normal distribution tables only covering values of *Z <* 6, *p* < 9.86 X 10^−10^_._ To calculate the random effects *p*-value, we computed a *t*-statistic with the following equation (*df* = *K*-1): $\frac{\bar{Z_{r}}}{\sqrt{\left(\frac{1}{K})(S_{\bar{Z_{r}}}^{2}\right)}}$. All analyses were performed according to guidelines specified in [65, 67].

Using a fixed effects approach, we calculated a weighted *r* effect size for each hypothesized variable across the three studies that represented the magnitude of the between-group differences on our focused tests. We found medium effect sizes for grateful, indebted, and moved, a small to medium effect size for emodiversity, and small effect sizes for uplifted and connected to others, such that participants in the gratitude conditions reported higher levels of these states than those in the comparison conditions. However, our 95% confidence interval for guilty included zero, indicating that our effect size was not significantly different from zero. In sum, across three studies, we found strong evidence that gratitude exercises elicit feelings of being grateful, moved, uplifted, and connected to others, but also indebted. That said, we did not find evidence for more noxious social negative emotions like guilt, embarrassment and shame in response to gratitude exercises.

## General discussion

Across three studies, gratitude exercises elicited a mix of socially-relevant pleasant and unpleasant states. In the first two studies, we compared gratitude inductions to positive emotion inductions (namely, relief) that were nonsocial and found that, as predicted, expressing or recalling gratitude elicited greater feelings of being grateful, moved, uplifted, connected, and indebted, but not more guilty. Thus, it is not just receiving any benefit—only a social benefit—that elicits this particular array of socially-relevant emotions. In our third study, we compared a gratitude exercise to another socially-oriented positive emotion exercise (i.e., writing about a kind act one performed for others), and found that, as predicted, the gratitude exercise promoted greater feelings of being grateful, moved, uplifted, indebted, and guilty, but not more embarrassed or ashamed. Thus, it is not just any social exchange—only the receipt of a benefit—that gives rise to this distinct set of socially-relevant emotions. Similarly, the gratitude exercise promoted greater feelings of being grateful, moved, uplifted, and indebted, but no greater guilt, than a neutral control condition.

In sum, across our comparison conditions in our three studies, we saw the uniqueness of gratitude take shape by comparing specific core features of gratitude to other exercises that include some, but not all, these core features. Despite minor differences, across three studies, we found compelling and consistent evidence that gratitude exercises produce a mixed emotional experience—one that is simultaneously uplifting and mildly uncomfortable (for a summary of effects across studies, see the meta-analysis in Table 4). Because gratitude exercises have been promoted as positive exercises that benefit relationships, health, work, and overall well-being if practiced regularly (e.g., [2, 68]), it is critical to understand the emotional landscape immediately following their practice, as well as the conditions under which gratitude exercises might be helpful for some individuals but backfire for others.

### Implications of a mixed emotional experience

People feel grateful when they recognize that someone has done something for them that they did not necessarily earn [7]. Thus, gratitude feels good, as recipients acknowledge that others care about them and they experience a sense of social belonging—but also mildly uncomfortable, as recipients may ponder whether they deserved the kind act or have not yet proven themselves worthy of it. Hence, they may also strive to live up to their benefactor’s support by paying back the kind act or paying it forward to restore social balance. In this way, gratitude may be particularly motivating, as it operates on the activating properties of both pleasant and unpleasant emotions.

Indeed, to the extent that positive emotions broaden people’s perspectives and stimulate them to build intellectual, social, and physical resources [69, 70], states like gratitude and elevation that draw attention to the goodness of others should be particularly motivating, as they prompt one to do good deeds or strive to become a better person [40]. Similarly, connectedness—another proximal outcome of gratitude inductions—could also serve as an important catalyst of improving oneself and one’s relationships [71, 72].

Of course, negative states also prompt action. Indeed, the desire to avoid or dispel negative emotions and outcomes is stronger than the desire to approach positive ones [73, 74], and people often change their behavior or attitudes to reduce uncomfortable discrepancies between them [75]. For example, if individuals feel undeserving of a favor or gift, they might be motivated to reduce their indebtedness or guilt by paying the deed forward or back or by doing something to prove themselves deserving of the deed (e.g., [76]; but see [77]). On the other hand, if the guilt or indebtedness is overwhelming, it could result in inaction, or worse, a pattern of rumination and worry [78]. Accordingly, gratitude exercises could be harmful under certain conditions or for certain individuals (e.g., those particularly prone to guilt) if they stimulate intense negative emotions.

If people were simply content and positive at all times, they would not strive to gain knowledge and skill at work, develop deeper relationships, or achieve healthier lifestyles. Perhaps gratitude exercises are effective for producing positive outcomes because they invigorate people with positive emotions and a sense of support from close others but still leave enough lingering unpleasant feelings to light a fire of change. Indeed, people who rate themselves on the highest rung of the happiness ladder are less successful in work, education, and political participation than their slightly less happy peers [79].

Notably, according to our cluster analyses, in all three studies, the gratitude groups were more likely than the comparison groups (i.e., two relief exercises, kindness, and neutral control) to experience high levels of pleasant socially-relevant emotions, as well as indebtedness. Furthermore, in Study 3, we found that the gratitude group was more likely than the kindness or neutral control groups to have an emotional profile with relatively high levels of pleasant emotions, as well as relatively high levels of indebtedness, guilt, embarrassment, and shame. This last finding represented close to half of gratitude letter writers, indicating that for some people—perhaps those already prone to guilt and shame—gratitude elicits relatively high levels of unpleasant emotions that could undermine the positive effects of the exercise or even harm well-being.

### The nature of indebtedness

Across the three studies, our most robust mean-level finding vis-a-vis an unpleasant state was that gratitude elicited more indebtedness than the comparison conditions. Although some individuals experienced guilt, embarrassment, and even shame after engaging in a gratitude exercise, indebtedness was the most consistently and strongly felt negative state among participants prompted to recall gratitude. Interestingly, although indebtedness is often experienced as distressing and uncomfortable, correlational patterns across all three studies demonstrated that it is also related to pleasant states like feeling grateful, happy, moved, and uplifted. However, in Studies 1 and 3 (and marginally in Study 2), indebtedness was also positively related to guilt, and in Study 3 (and marginally in Study 2), it was also positively related to other negative states like worry and depression (see also [50, 53]).

Relative to indebtedness, unpleasant emotions such as worry and shame show a decidedly more negative profile, with stronger correlations with other negative emotions and with significant inverse relations to positive emotions like happiness and joy. However, although seemingly not as intensely unpleasant as worry and shame, we argue that indebtedness is a mild negative state that prompts an individual to act to restore balance in a social relationship. An individual feels indebted when another person deems him or her worthy of time and effort in the form of a kind act, gift, or favor. Accordingly, feelings of indebtedness involve asking oneself whether one deserved the act and how to pay it back. Thus, indebted individuals are not typically paralyzed by feelings of shame, worry, and unhappiness, but simply feel the nagging sense that they need to restore balance to a relationship. Correlational analyses reveal that this nagging sense can coincide with both positive and negative emotions, but does not detract from or is neutralized by positive emotions (as evidenced by the absence of negative correlations between indebtedness and any positive emotion). Thus, indebtedness appears to be a somewhat uncomfortable and unpleasant but ultimately beneficial state—one that has potential to drive positive actions that strengthen relationships, improving well-being in the long-term. Indeed, past research has demonstrated that feeling indebted has more positive consequences than feeling obligated: Feeling indebted, like feeling grateful, was related to prosocial action tendencies, whereas feeling obligated was related to antisocial tendencies (e.g., rejecting or avoiding one’s benefactor and withdrawing from one’s environment [50]).

### Potential downsides to gratitude exercises

While expressing or recalling gratitude for a gift or favor, people may decide that they did not deserve the kind act or, even worse, that they are particularly unworthy. In these instances, in addition to feeling indebted, one may also feel guilt, embarrassment, or shame. We originally predicted that guilt, embarrassment, and shame would be common responses to a gratitude exercise, but we only found a significant effect on guilt in one out of our three studies and no effect on embarrassment or shame in the one study in which those two variables were included. Based on our cluster analyses from Study 3, it appears that these noxious cousins of indebtedness are sometimes felt after engaging in a gratitude exercise, but likely only by a subset of individuals.

Past research implicates groups that might be more likely to experience high levels of both pleasant and unpleasant emotions, or possibly even a preponderance of unpleasant emotions, in response to a gratitude exercise. For example, one study found that South Korean students, unlike their American counterparts, did not show improved well-being after writing gratitude letters [13]. Although not tested in that study, the authors concluded that South Koreans likely experienced more of the conflicting feelings we found in the current studies, such as indebtedness and guilt, compared to Americans. Consistent with this notion, some collectivist cultures are relatively uncomfortable with seeking social support from close others, are prone to guilt, and desire to avoid worrying friends and family and “putting others out” (e.g., [80–84]). Indeed, East Asians are more likely than North Americans to turn down even small gifts from acquaintances to avoid feeling indebted [85].

In light of this reasoning, we predicted that South Koreans in Study 3 would feel more unpleasant emotions (and therefore more mixed emotions overall) in the gratitude condition than in the kindness or control conditions. Contrary to this prediction, our intended neutral control condition actually evoked more unpleasant emotions for South Koreans than the gratitude or kindness conditions. Perhaps recounting what they did over the past week reminded South Koreans (who were enrolled at a highly competitive university) of what they had failed to accomplish, thus stimulating discomfort. Alternatively, both the gratitude and kindness conditions may have alleviated negative feelings the participants were already experiencing and the control comparison simply sustained their unpleasant mood. Because we only collected the emotion measures post-test—a limitation of our studies—we cannot conclude whether these emotional states increased or decreased over time. Future researchers would do well to administer both baseline and post-test measures to explore the direction of emotion shifts across conditions as a result of the writing exercises.

In addition, given the success of gratitude interventions in raising well-being among healthy populations, researchers have theorized that they could also play an important role in treatment for clinical disorders such as depression and anxiety [86–88]. However, our findings suggest that to the extent that depressed or anxious individuals are particularly sensitive to feelings of indebtedness, guilt, embarrassment, or shame, gratitude interventions could backfire in this population. For example, recent evidence has demonstrated that people who are relatively high in social anxiety, public self-consciousness, and prevention-focus are also high in trait indebtedness [47–48].

To date, the literature on gratitude interventions in subclinical depressed samples is mixed. Although some studies have found that gratitude interventions alleviate depression in mildly or moderately depressed samples (e.g., [11, 20]) and may even be more effective among those with higher levels of depression [25], one study with a mildly depressed sample found that writing gratitude letters actually diminished immediate well-being [89]. Because over half of the individuals who seek happiness-increasing strategies online (including gratitude interventions) meet the diagnostic criteria for clinical depression [90], further research is needed to uncover the circumstances under which the mixed emotional nature of gratitude exercises could produce adverse consequences.

### Future directions and conclusions

The current studies are the first to our knowledge to explore the array of discrete proximal emotions felt after engaging in gratitude exercises and to provide evidence that such exercises can produce both pleasant and unpleasant states. However, these studies prompt a host of unanswered questions, including the issues of individual differences and downstream consequences already raised. Another intriguing question is whether particular targets of gratitude or particular types of kind acts might produce unique blends of pleasant and unpleasant emotions. For example, expressing gratitude for relatively large kind acts that require more effort on the part of the benefactor or recalling an act from benefactors of higher status may lead participants to feel relatively more indebted, guilty, or uncomfortable. Similarly, recalling a kind act that can never be repaid may produce more of those unpleasant feelings than recalling one that can readily be repaid. One approach to addressing this question would be to experimentally manipulate the instructions, such that participants are prompted to write gratitude letters for kindnesses that are large versus small, easily repaid versus not, or for benefactors that are high status versus peers. Additionally, future research could explore how gratitude might influence other social emotions not tested in the current studies, such as admiration, respect, and love, to understand the full array of emotions evoked by gratitude exercises.

Another promising approach is to compare different types of gratitude exercises beyond the two tested here. For example, the exercise of counting one’s blessings (e.g., for a sunny day or a smile from a stranger) could be less aversive for depressed individuals or for certain cultural groups than writing a full-fledged gratitude letter [91]. Some individuals may feel comfortable writing about blessings, which can be found in many small day-to-day encounters, but experience discomfort writing about the types of larger deeds towards which gratitude letters are typically targeted. Future studies could test whether counting blessings stimulates similar levels of indebtedness or guilt as writing letters. Relatedly, given that our intended neutral control task was potentially emotionally evocative for South Koreans, future studies should continue to test and refine a variety of neutral and emotionally-evocative comparison conditions to further illuminate the effect of gratitude expression compared to other neutral and emotion exercises.

Gratitude expression has been promoted throughout history across the world’s religious and cultural traditions [1], and contemporary society betrays a growing interest in gratitude interventions as a panacea for everyday struggles and major stresses alike. Accordingly, we argue that understanding people’s emotional state immediately after engaging in a gratitude exercise—and ultimately how it could affect future behavior—is crucial. In the current studies, we found evidence that gratitude exercises feel both pleasant (as many other studies have found) *and* mildly unpleasant. Furthermore, we speculate that a mixed emotional experience, rather than a purely positive one, might be particularly motivating. Indeed, it may be this bittersweet state and the behaviors it elicits that explain why gratitude exercises lead to downstream positive outcomes (e.g., prosociality, health-promoting behavior), lending support to the age-old wisdom that gratitude is indeed a virtue.

## Supporting information

S1 File Supporting information. This file includes instructions for all conditions, all measures, and tables with additional descriptive statistics and analyses. In addition to the analyses referenced in the main manuscript, we also included condition by sex analyses in Tables E and I for Studies 1 and 2, respectively, and condition by sex by culture analyses in Table U for Study 3.
(PDF)
