# Supplementary material for: The proximal experience of gratitude
Source: PLoS One. 2017 Jul 7;12(7):e0179123. doi: 10.1371/journal.pone.0179123 (PMC5501400; doi:10.1371/journal.pone.0179123)
Supplement: S1 File — This file includes instructions for all conditions, all measures, and tables with additional descriptive statistics and analyses. In addition to the analyses referenced in the main manuscript, we also included condition by sex analyses in Tables E and I for Studies 1 and 2, respectively, and condition by sex by culture analyses in Table U for Study 3. (PDF) [file pone.0179123.s001.pdf]

Supporting information (S1 File)

The Proximal Experience of Gratitude

Kristin Layous[1]

Kate Sweeny[2]

Christina Armenta[2]

Soojung Na[3]

Incheol Choi[3]

Sonja Lyubomirsky[2]

[1]California State University, East Bay

[2]University of California, Riverside

[3]Seoul National University

Author Note

Correspondence should be addressed to Kristin Layous at the Department of Psychology, California State University, East Bay, 25800 Carlos Bee Blvd, Hayward, CA 94542-3091. E-mail: kristin.layous@csueastbay.edu.

Study 1

Recruitment Text
The purpose of this study is to learn about everyday experiences. You will recall recent life experiences and then complete several questionnaires about those experiences. The survey should take about 15 minutes to complete.

Experimental Instructions:

Near-Miss Relief
Think of a time during the last month when you narrowly avoided a bad outcome and felt RELIEF as a result. For example...

- Maybe you thought something was lost, and then you found it
- Maybe you almost had a car accident or almost injured yourself, but avoided it at the last minute
- Maybe you almost did something embarrassing or damaging in some way, but avoided it at the last minute
- Maybe you thought something bad had happened, but then found out it hadn't happened

Take a few moments to recall your experience of RELIEF in as much detail as you can. Now, write about the experience, including what almost went wrong, how it was avoided, and how you felt throughout the experience.

Please do not provide identifying information such as your name, place of work etc. If these details are important to the story, just put "XXXX" in place of the identifying detail.

Task-Completion Relief
Think of a time during the last month when you finished an unpleasant task and felt RELIEF as a result. For example...

- Maybe you finished a dreaded exam or project
- Maybe you got through a long or difficult day at work or school
- Maybe you got through a presentation, conversation, or confrontation that you were worried about

Take a few moments to recall your experience of RELIEF in as much detail as you can. Now, write about the experience, including what almost went wrong, how it was avoided, and how you felt throughout the experience.

Please do not provide identifying information such as your name, place of work etc. If these details are important to the story, just put "XXXX" in place of the identifying detail.

Gratitude
Think of a time during the last month when someone did something for you for which you were truly GRATEFUL. For example...

- Maybe they took care of you when you were sick
- Maybe they helped you prepare for a stressful exam
- Maybe they did something to brighten your day out of the blue

Take a few moments to recall your experience of GRATITUDE in as much detail as you can. Now, write about the experience, including who that person is, what they did for you, and how you felt throughout the experience.

Please do not provide identifying information such as your name, place of work etc. If these details are important to the story, just put "XXXX" in place of the identifying detail.

Outcome Measures:
The following scale consists of a number of words that describe different feelings and emotions. Read each item and then select the response that best indicates how you are feeling RIGHT NOW.

Right now, I feel... (7-point scale; anchors: Not at all, Very Slightly, Somewhat, Moderately, Much, Very Much, Extremely)

- Moved
- Uplifted
- Guilty
- Optimistic about humanity
- A warm feeling in your chest
- Humbled
- Indebted (feeling like you need to repay another for their actions that benefitted you)
- A desire to help others
- A desire to become a better person
- Grateful
- Happy
- Worried/anxious
- Pleased
- Relieved
- Angry
- Frustrated
- Depressed/blue
- Unhappy
- Inspired
- Scared
- Upset
- Nervous

For this set of questions, please focus on the FEELING of relief/gratitude (depending on condition) you had during the experience you wrote about (5-point scale; anchors: Strongly disagree, Disagree, Neither agree not disagree, Agree, Strongly agree)

- The feeling of relief/gratitude was mild
- The feeling of relief/gratitude was intense
- The feeling of relief/gratitude came on suddenly
- The feeling of relief/gratitude developed gradually
- The feeling of relief/gratitude was only positive
- The feeling of relief/gratitude was only negative
- The feeling of relief/gratitude was a mix of positive and negative
- The feeling of relief/gratitude made me feel more connected to other people
- The feeling of relief/gratitude made me feel isolated from other people.
- The feeling of relief/gratitude made me feel like doing something for someone else.
- The feeling of relief/gratitude made me want to be a better person.
- I felt energetic when the relief/gratitude hit me.
- I felt calm when the relief/gratitude hit me.

The following questions ask about your thoughts you might have had during your experience. Continue to focus on the period of time WHILE you were experiencing this feeling (7-point scale; anchors: 1 = Not at all, 4 = Somewhat, 7 = Very Much).

- To what extent were you thinking about how things could have turned out differently?
- To what extent were you thinking about how things could have turned out BETTER?
- To what extent were you thinking about how things could have been WORSE?

Demographics

Study 2

Recruitment Text
The purpose of this study is to learn about every day experiences. You will recall recent life experiences and then complete several questionnaires about those experiences. The survey should take about 15 minutes to complete.

Experimental Instructions:

Gratitude Letter
Please take a moment to think back over the past several years of your life and remember an instance when someone did something for you for which you are extremely grateful. For example, think of the people – parents, relatives, friends, teachers, coaches, teammates, employers, and so on – who have been especially kind to you but have never heard you express your gratitude.

Now, for the next 10 minutes, write a letter to one of these individuals. Use the instructions below to help guide you through this process:

1. Use whatever letter format you like, but remember to write as though you are directly addressing the individual you are grateful to. If it is helpful to head the letter “Dear so-and-so,” or end with “Sincerely, XXX,” feel free to do so.

2. Do not worry about perfect grammar and spelling.

3. Describe in specific terms why you are grateful to this individual and how the individual’s behavior affected your life.

4. Describe what you are doing now and how you often remember their efforts.

5. Remember: Anything you write will remain strictly confidential. Although you are welcome to show or give this letter to anyone you please, for the purposes of this study, the letter you write is a private document in which you can express your gratitude freely without intent to deliver it to anyone. Should an experimenter read this entry in the future, it will be identifiable only by a subject number and not by a name.

***All of the same measures were used as in Study 1**

Study 3

Recruitment Text
This is a study about life experiences. In this study, you will be asked to write about your life experiences and then complete online questionnaires about your thoughts and emotions.

If you would like to sign up for this study, please visit the following website to create a unique username and password: www.modernresearch.org. You will be asked for your name and email address, but this information will be kept separate from your responses to the study. If you have any questions regarding the study, feel free to email pplab.ucr@gmail.com.

Experimental Manipulations:

Gratitude
Please take a moment to think back over the past several years of your life and remember an instance when someone did a kind act (or acts) for you for which you are extremely grateful. For example, think of the people – parents, children, spouses/partners, relatives, friends, neighbors, teachers, employers, and so on – who have been especially generous and thoughtful towards you. Now for the next 5 minutes, write about how that person helped you. Use the instructions below to help guide you through this process:

1. Do not worry about perfect grammar and spelling.
2. Describe in specific terms the kind act this person bestowed upon you and how the kind act affected your life.
3. Describe what you are doing now and how you often remember their efforts.
4. Remember: Anything you write will remain strictly confidential. Should an experimenter read this entry in the future, it will be identifiable only by a subject number and not by a name.

Kindness
Please take a moment to think back over the past several years of your life and remember an instance when you did a kind act (or acts) for someone. For example, think of the family members, friends, fellow students, or fellow community members with whom you have interacted and to whom you’ve been especially generous or thoughtful. Now for the next 5 minutes, write about who you helped and why. Use the instructions below to help guide you through this process:

1. Do not worry about perfect grammar and spelling.
2. Describe in specific terms the kind act or acts you performed and how it affected the person’s life.
3. Describe what they are doing now and how often you think about them.

4. Remember: Anything you write will remain strictly confidential. Should an experimenter read this entry in the future, it will be identifiable only by a subject number and not by a name.

Control
Please take a moment to think about what you did during the last 7 days. That is, create a mental outline of what you did during that time. Now, for the next 5 minutes, please write out these activities in a list format. Be as detail-oriented as possible, but try to leave out emotions, feelings, or opinions pertaining to your plans. In other words, focus on exactly what you did.

Finally, as you write, don't worry about perfect grammar and spelling, and remember that anything you write will remain strictly confidential. Should an experimenter read this entry in the future, it will be identifiable only by a participant number and not by a name.

Post-Manipulation Measures
For the purposes of other research questions about measurement issues in positive affect, we included two affect scales (along with our other measures), both of which the current study draws from to explore as similar emotions as possible to the other studies.

Elevation Scale (based on the work by John Haidt (2003a, 2003b); measure from Schnall, Roper, and Fessler, 2012)
*We added guilty, indebted, and grateful to explore our other hypotheses

While engaging in the writing task, to what degree did you feel each of the following emotions? (1 = *didn't feel at all,* 4 = *felt moderately,* 7 = *felt very strongly*)

1. Moved
2. Uplifted
3. Guilty
4. Optimistic about humanity
5. A warm feeling in your chest
6. Indebted (feeling like you need to repay another for their actions that benefitted you)
7. A desire to help others
8. A desire to become a better person
9. Grateful
10. Happy

Affect-Adjective Scale (Diener & Emmons, 1985)
*Note: For separate research questions about measurement issues in positive and emotions, we included two measures of positive emotions, the Affect-Adjective Scale (Diener & Emmons, 1985) and the Modified Differential Emotions Scale (mDES; Fredrickson, Tugade, Waugh, & Larkin, 2003; see all measures in Supplemental Materials). Most of the emotions from the first three studies are reflected in the elevation and Affect-Adjective scales, but we drew *embarrassed* and *ashamed* from the mDES, which were actually listed as "embarrassed, self-conscious, blushing" and "ashamed, humiliated, and disgraced," respectively. We converted those items from a 5-point scale to a 7-point scale to match the other emotions.

Using the 7-point scale below, please indicate the extent to which you feel this way RIGHT NOW (*Not at all, Very slightly, Somewhat, Moderately, Much, Very Much).*

Worried/Anxious
Pleased
Angry
Frustrated
Depressed/Blue
Joyful
Unhappy
Enjoyment/Fun

GQ-6 (adapted to reflect state gratitude; McCullough, Emmons, & Tsang, 2002)
Please indicate the extent to which you agree with each statement below (1 = *strongly disagree*, 2 = *disagree*, 3 = *slightly disagree*, 4 = *neutral*, 5 = *slightly agree*, 6 = *agree*, 7 = *strongly agree*).

1. Right now I feel that I have much in life to be thankful for.
2. If I had to list everything that I was grateful for right now, it would be a very long list.
3. When I look at the world, I don’t see much to be grateful for right now (reverse-scored).
4. I am grateful to a wide variety of people currently in my life.
5. Right now, I find myself more able to appreciate the people, events, and situations that have been part of my life history.
6. Right now, I find it difficult to feel grateful to something or someone (reverse-scored)

Just Connectedness Scale of Balanced Measure for Psychological Needs (state version; Sheldon & Hilpert, 2012)
Please read each of the following statements carefully. Select the point on the scale that represents how true each statement is for you RIGHT NOW (1 = *no agreement*, 3 = *some agreement*, 5 = *much agreement*)

1. I feel a sense of contact with people who care for me, and whom I care for.
2. I feel lonely (reverse-scored)
3. I feel close and connected with other people who are important to me.
4. I feel unappreciated by one or more important people (reverse-scored)
5. I feel a strong sense of intimacy with the people I spend time with.
6. I am having disagreement or conflicts with people I usually get along with (reverse-scored)

Modified Differential Emotions Scale (mDES; Frederickson, Cohn, Coffey, Pek, & Finkel, 2008)
For each of the following items, please click the item that best describes the greatest amount of each emotion you feel RIGHT NOW (*Never, Hardly, Some of the time, Often, All the time*).

1. I have felt amused, fun-loving, silly
2. I have felt angry, irritated, annoyed
3. I have felt ashamed, humiliated, disgraced
4. I have felt awe, wonder, amazement
5. I have felt contemptuous, scornful, disdainful

6. I have felt content, serene, peaceful
7. I have felt disgust, distaste, revulsion
8. I have felt embarrassed, self-conscious, blushing
9. I have felt glad, happy, joyful
10. I have felt grateful, appreciative, thankful
11. I have felt hopeful, optimistic, encouraged
12. I have felt interested, alert, curious
13. I have felt love, closeness, trust
14. I have felt proud, confident, self-assured
15. I have felt repentant, guilty, blameworthy
16. I have felt sad, downhearted, unhappy
17. I have felt scared, fearful, afraid
18. I have felt attracted to someone, flirtatious
19. I have felt surprised, amazed, astonished
20. I have felt sympathy, concern, compassion

State-Trait Anxiety Inventory (STAI; shortened version; validated in van der Bij, de Weerd, Cikot, Steegers, & Braspenning, 2003).

A number of statements which people have used to describe themselves are given below. Read each statement and use the following scale to indicate how you feel right now, at this very moment. There are no right or wrong answers. Do not spend too much time on any one statement, but give the answer which seems to best describe your present feelings (*Not at all, Somewhat, Moderately, Very much)*

1. I feel calm.
2. I am tense.
3. I feel upset.
4. I am relaxed.
5. I feel content.
6. I am worried.

Subjective Happiness Scale (Lyubomirsky & Lepper, 1999)
Satisfaction With Life Scale (Diener, Emmons, Larsen, & Griffin, 1985)
Balanced Measure of Psychological Needs (Sheldon & Hilpert, 2012)

The State-Trait Anxiety Inventory, Subjective Happiness Scale, and Satisfaction With Life Scale, and the Balanced Measure of Psychological Needs did not show any differences across conditions, all Fs < 1.19.

Demographics

Table A

Contrast Analyses on Elevation Composite

Study	*df*	$t_{contrast}$	*p*	*r* 95% CI[]
Study 1				
Contrast 1	134	2.06	.04	.18 [.01, .33]
Contrast 2	102	2.19	.03	.21 [.02, .39]
Contrast 3	82	1.29	.20	.14 [-.07, .34]
Study 2				
Contrast 1	127	2.31	.02	.20 [.03, .36]
Contrast 2	93	1.92	.06	.20 [-.01, .38]
Contrast 3	77	2.06	.04	.23 [.01, .43]
Study 3				
Contrast 1	427	3.93	<.001	.19 [.09, .28]
Contrast 2	291	15.76	<.001	.68 [.61, .74]
Contrast 3	280	11.55	<.001	.57 [.48, .64]

Note: The elevation composite included five items: Moved, uplifted, optimistic about humanity, a warm feeling in your chest, a desire to help others, and a desire to become a better person (Schnall, Roper, & Fessler, 2012; (7-point scale indicating the degree to which they felt each of the above items right now; anchors: Not at all, Very Slightly, Somewhat, Moderately, Much, Very Much, Extremely). To focus the main manuscript on emotions, we only presented “moved” and “uplifted” to represent this elevation composite. We include the results on the full composite here for interested readers.

Contrast Weights:
Study 1: *Contrast 1:* Gratitude Experience (+1), Gratitude Letter (+1), Near-Miss Relief (-2); *Contrast 2:* Gratitude Experience (+1), Gratitude Letter (0), Near-Miss Relief (-1); *Contrast 3*: Gratitude Experience (0), Gratitude Letter (+1), Near-Miss Relief (-1).

Study 2: *Contrast 1*: Gratitude Experience (+2), Near-Miss Relief (-1), Task-Completion Relief (-1); *Contrast 2*: Gratitude Experience (+1), Near-Miss Relief (-1), Task-Completion (0); *Contrast 3:* Gratitude Experience (+1), Near-Miss Relief (0), Task-Completion Relief (-1).

Study 3: *Contrast 1:* Gratitude (+1), Kindness (-1), Control (0); *Contrast 2*: Gratitude (+1), Kindness (0), Control (-1); *Contrast 3:* Gratitude (0), Kindness (+1), Control (-1).

Table B

Bivariate Correlations Among Variables in Study 1

**Variable**	**1**	**2**	**3**	**4**	**5**	**6**	**7**	**8**	**9**	**10**	**11**	**12**	**13**	**14**	**15**	**16**	**17**	**18**	**19**
1. Grateful	--																		
2. Relieved	.52	--																	
3. Indebted	.42	.32	--																
4. Guilty	-.01	.10	.20	--															
5. Moved	.39	.36	.46	.23	--														
6. Uplifted	.46	.36	.47	.17	.68	--													
7. Connected to others	.34	.26	.26	-.09	.38	.37	--												
8. Happy	.64	.48	.35	-.06	.28	.38	.27	--											
9. Worried/anxious	-.26	-.11	-.03	.37	-.08	-.15	-.25	-.37	--										
10. Angry	-.31	-.03	-.03	.51	.05	-.06	-.14	-.34	.61	--									
11. Frustrated	-.30	-.02	-.14	.44	.01	-.12	-.17	-.39	.66	.83	--								
12. Depressed/blue	-.33	-.12	-.09	.36	-.10	-.12	-.21	-.44	.63	.66	.77	--							
13. Joyful	.61	.52	.23	-.10	.32	.37	.38	.68	-.33	-.25	-.27	-.29	--						
14. Nervous	-.17	.05	.05	.45	.12	-.01	-.09	-.21	.63	.56	.63	.59	-.27	--					
15. Inspired	.56	.42	.36	-.001	.47	.49	.28	.52	-.12	-.15	-.18	-.17	.58	-.03	--				
16. Scared	-.01	.09	.02	.46	.22	.01	.05	-.10	.52	.54	.58	.42	-.07	.60	.06	--			
17. Upset	-.33	-.08	-.08	.43	.05	-.14	-.18	-.41	.75	.75	.77	.76	-.34	.77	-.16	.62	--		
18. Unhappy	-.21	-.05	-.02	.36	.02	-.05	-.11	-.31	.62	.67	.73	.70	-.35	.59	-.12	.52	.73	--	
19. Pleased	.55	.55	.32	-.09	.41	.39	.33	.50	-.28	-.21	-.26	-.30	.57	-.20	.43	-.04	-.29	-.24	--

Note: Correlations are all based on at least 133 participants. Correlations .17 and above are significant at $p \leq .05$. Correlations .22 and above are significant at $p \leq .01$. Correlations .28 and above are significant at $p \leq .001$.

Table C

Cluster Centroids for Study 1

Item	*M* (*SD*) across conditions	Cluster 1 (*n* = 85)	Cluster 2 (*n* = 49)
	All Participants		
Grateful	5.64 (1.41)	6	5
Indebted	4.21 (1.92)	5	3
Guilty	1.44 (1.99)	2	2
Moved	4.07 (1.82)	5	2
Uplifted	4.29 (1.75)	5	3
Connected	4.91 (1.60)	5.43	4

Note: Centroids are the average of the scores for specific element of the cluster (e.g., Grateful or Moved) for that cluster (e.g., for Cluster 1). The centroid values should be interpreted with the means of the sample in mind. For example, similar to past research (Diener & Diener, 1996; Diener, Kanazawa, Suh, & Oishi, 2014), people reported higher positive emotions than negative emotions on average, so all clusters contain centroids reflecting higher positive emotions than negative emotions. However, because the mean for indebtedness across the sample is 4.21 (on a 7-point scale), we can interpret that participants in Cluster 1 have high levels of indebtedness relative to the rest of the sample.

Table D

Table of Counts by Condition and Cluster Assignment for Study 1

Cluster Assignment	Gratitude Experience	Gratitude Letter	Near-Miss Relief	Total
Cluster 1	33 (63.46%)	26 (78.79%)	26 (53.06%)	85
Cluster 2	19 (36.54%)	7 (21.21%)	23 (46.94%)	49
Total	52	33	49	134

Note: Percentages represent proportion of each condition in each cluster. Participants per condition are lower than in other analyses because four participants were listwise deleted for failing to answer at least one of the six variables.

Table E

Effects of Sex and Condition on Dependent Variables in Study 1

Source	*df*	*F*	*p*	Condition	*M(SE)*	
Grateful					Men	Women
Condition	2	2.14	.12	Gratitude Experience:	5.71 (0.27)	5.77 (0.30)
Sex	1	0.12	.73	Gratitude Letter:	5.96 (0.30)	6.00 (0.43)
Condition X Sex	2	0.03	.98	Near-Miss Relief:	5.24 (0.28)	5.41 (0.30)
Error	124					
Relieved					Men	Women
Condition	2	3.10	.05	Gratitude Experience:	4.93 (0.34)	3.46 (0.39)
Sex	1	3.48	.07	Gratitude Letter:	5.00 (0.40)	4.55 (0.55)
Condition X Sex	2	2.17	.12	Near-Miss Relief:	5.08 (0.37)	5.14 (0.39)
Error	122					
Indebted					Men	Women
Condition	2	4.29	.02	Gratitude Experience:	4.46 (0.35)	4.55 (0.40)
Sex	1	2.13	.15	Gratitude Letter:	4.86 (0.40)	4.36 (0.56)
Condition X Sex	2	1.16	.32	Near-Miss Relief:	4.08 (0.37)	3.00 (0.40)
Error	124					
Guilty					Men	Women
Condition	2	1.72	.18	Gratitude Experience:	1.89 (0.28)	1.73 (0.31)
Sex	1	2.37	.13	Gratitude Letter:	2.05 (0.31)	1.46 (0.44)
Condition X Sex	2	0.24	.79	Near-Miss Relief:	2.52 (0.29)	2.05 (0.31)
Error	124					
Moved					Men	Women
Condition	2	5.09	.01	Gratitude Experience:	4.46 (0.33)	3.96 (0.38)
Sex	1	1.26	.26	Gratitude Letter:	4.59 (0.38)	4.73 (0.53)
Condition X Sex	2	0.54	.58	Near-Miss Relief:	3.76 (0.35)	3.05 (0.38)
Error	124					
Uplifted					Men	Women
Condition	2	5.06	.01	Gratitude Experience:	4.46 (0.32)	4.18 (0.36)
Sex	1	0.01	.92	Gratitude Letter:	4.91 (0.37)	5.18 (0.51)
Condition X Sex	2	0.29	.75	Near-Miss Relief:	3.72 (0.34)	3.82 (0.36)
Error	123					
Connected to others					Men	Women
Condition	2	5.02	.01	Gratitude Experience:	5.61 (0.29)	5.16 (0.29)
Sex	1	1.21	.27	Gratitude Letter:	5.16 (0.33)	4.96 (0.33)
Condition X Sex	2	0.07	.93	Near-Miss Relief:	4.54 (0.31)	4.27 (0.33)
Error	123					
Happy					Men	Women
Condition	2	0.57	.57	Gratitude Experience:	5.57 (0.28)	5.55 (0.31)
Sex	1	0.07	.79	Gratitude Letter:	5.73 (0.31)	5.73 (0.44)
Condition X Sex	2	0.05	.95	Near-Miss Relief:	5.46 (0.30)	5.27 (0.31)
Error	123					
Worried/anxious					Men	Women
Condition	2	1.28	.28	Gratitude Experience:	2.00 (0.31)	2.14 (0.35)
Sex	1	1.08	.30	Gratitude Letter:	2.14 (0.35)	2.82 (0.49)
Condition X Sex	2	0.33	.72	Near-Miss Relief:	2.52 (0.33)	2.64 (0.35)
Error	124					
Angry					Men	Women
Condition	2	1.44	.24	Gratitude Experience:	1.64 (0.25)	1.36 (0.28)
Sex	1	2.21	.14	Gratitude Letter:	1.64 (0.28)	1.55 (0.39)
Condition X Sex	2	0.55	.58	Near-Miss Relief:	2.28 (0.26)	1.59 (0.28)
Error	124					

Table E continued

Source	*df*	*F*	*p*	Condition	*M(SE)*	
Frustrated					Men	Women
Condition	2	1.23	.30	Gratitude Experience:	1.96 (0.30)	1.77 (0.34)
Sex	1	0.74	.39	Gratitude Letter:	1.96 (0.34)	1.91 (0.48)
Condition X Sex	2	0.22	.80	Near-Miss Relief:	2.60 (0.32)	2.09 (0.34)
Error	124					
Depressed/blue					Men	Women
Condition	2	1.47	.24	Gratitude Experience:	1.64 (0.29)	1.86 (0.33)
Sex	1	0.60	.44	Gratitude Letter:	2.05 (0.33)	1.73 (0.47)
Condition X Sex	2	0.80	.45	Near-Miss Relief:	2.56 (0.31)	2.00 (0.33)
Error	124					
Joyful					Men	Women
Condition	2	1.55	.22	Gratitude Experience:	4.75 (0.31)	4.46 (0.35)
Sex	1	0.10	.75	Gratitude Letter:	5.05 (0.35)	5.36 (0.49)
Condition X Sex	2	0.41	.66	Near-Miss Relief:	4.76 (0.32)	4.46 (0.35)
Error	124					
Nervous					Men	Women
Condition	2	2.29	.11	Gratitude Experience:	1.82 (0.24)	1.46 (0.27)
Sex	1	5.01	.03	Gratitude Letter:	2.09 (0.27)	1.64 (0.39)
Condition X Sex	2	0.30	.74	Near-Miss Relief:	2.58 (0.26)	1.82 (0.27)
Error	123					
Inspired					Men	Women
Condition	2	4.59	.01	Gratitude Experience:	4.54 (0.34)	4.00 (0.38)
Sex	1	0.03	.88	Gratitude Letter:	5.05 (0.39)	5.64 (0.54)
Condition X Sex	2	0.96	.39	Near-Miss Relief:	4.08 (0.36)	4.18 (0.38)
Error	123					
Scared					Men	Women
Condition	2	0.80	.45	Gratitude Experience:	2.25 (0.29)	1.50 (0.33)
Sex	1	0.83	.36	Gratitude Letter:	1.96 (0.33)	2.36 (0.47)
Condition X Sex	2	1.32	.27	Near-Miss Relief:	2.48 (0.31)	2.05 (0.33)
Error	124					
Upset					Men	Women
Condition	2	1.43	.24	Gratitude Experience:	1.61 (0.28)	1.86 (0.31)
Sex	1	0.11	.74	Gratitude Letter:	1.82 (0.31)	1.82 (0.44)
Condition X Sex	2	0.88	.42	Near-Miss Relief:	2.48 (0.29)	1.96 (0.31)
Error	124					
Unhappy					Men	Women
Condition	2	1.33	.27	Gratitude Experience:	1.75 (0.28)	2.05 (0.31)
Sex	1	0.14	.71	Gratitude Letter:	1.68 (0.31)	1.91 (0.44)
Condition X Sex	2	0.42	.66	Near-Miss Relief:	2.40 (0.29)	2.18 (0.31)
Error	124					
Pleased					Men	Women
Condition	2	0.18	0.84	Gratitude Experience:	4.93 (0.32)	4.55 (0.36)
Sex	1	0.80	0.37	Gratitude Letter:	4.96 (0.36)	4.73 (0.51)
Condition X Sex	2	0.03	0.97	Near-Miss Relief:	4.72 (0.34)	4.50 (0.36)
Error	124					
Positive Emotion Composite					Men	Women
Condition	2	2.77	.07	Gratitude Experience:	5.00 (0.22)	4.56 (0.25)
Sex	1	0.62	.43	Gratitude Letter:	5.14 (0.24)	5.21 (0.35)
Condition X Sex	2	0.45	.64	Near-Miss Relief:	4.60 (0.23)	4.46 (0.25)
Error	124					

Table E continued

Source	*df*	*F*	*p*	Condition	*M(SE)*	
Negative Emotion Composite					Men	Women
Condition	2	1.11	.33	Gratitude Experience:	2.10 (0.21)	2.03 (0.23)
Sex	1	1.22	.27	Gratitude Letter:	2.22 (0.23)	2.16 (0.33)
Condition X Sex	2	0.61	.55	Near-Miss Relief:	2.65 (0.22)	2.14 (0.23)
Error	124					
Emodiversity: All emotions					Men	Women
Condition	2	1.14	.32	Gratitude Experience:	2.11 (0.06)	1.98 (0.06)
Sex	1	2.73	.10	Gratitude Letter:	2.11 (0.07)	2.13 (0.09)
Condition X Sex	2	0.77	.47	Near-Miss Relief:	2.09 (0.06)	1.93 (0.06)
Error	118					
Emodiversity: Social emotions					Men	Women
Condition	2	3.82	.03	Gratitude Experience:	0.89 (0.16)	0.84 (0.19)
Sex	1	1.91	.17	Gratitude Letter:	1.20 (0.19)	0.95 (0.26)
Condition X Sex	2	0.38	.68	Near-Miss Relief:	1.53 (0.18)	1.17 (0.19)
Error	123					

Note: No significant condition by interaction effects in Study 1.

Table F

Bivariate Correlations Among Variables in Study 2

Variable	1	2	3	4	5	6	7	8	9	10	11	12	13	14	15	16	17	18	19
1. Grateful	--																		
2. Relieved	.55	--																	
3. Indebted	.48	.16	--																
4. Guilty	.01	-.02	.15	--															
5. Moved	.54	.33	.45	.28	--														
6. Uplifted	.58	.40	.53	.13	.73	--													
7. Connected to others	.33	.13	.36	-.10	.27	.30	--												
8. Happy	.74	.50	.29	-.09	.43	.50	.20	--											
9. Worried/anxious	-.05	-.09	.16	.60	.10	.02	-.06	-.22	--										
10. Angry	-.11	.09	.09	.63	.16	.02	-.04	-.24	.72	--									
11. Frustrated	-.05	.07	.07	.64	.18	-.01	-.02	-.23	.66	.86	--								
12. Depressed/blue	-.05	-.01	.15	.54	.15	.01	.03	-.29	.66	.70	.79	--							
13. Joyful	.63	.44	.37	-.02	.54	.54	.433	.69	-.13	-.03	-.06	-.20	--						
14. Nervous	-.02	.05	.15	.57	.22	.06	.02	-.20	.70	.71	.78	.72	-.06	--					
15. Inspired	.59	.36	.43	.08	.62	.62	.38	.56	-.04	.03	.04	-.03	.75	.09	--				
16. Scared	.03	.13	.16	.56	.25	.09	.09	-.11	.61	.67	.70	.65	.08	.80	.21	--			
17. Upset	.01	.07	.14	.61	.29	.10	.03	-.12	.68	.75	.78	.67	.01	.89	.10	.80	--		
18. Unhappy	-.04	-.003	.15	.67	.20	.07	.03	-.26	.75	.79	.77	.78	-.09	.71	.06	.72	.66	--	
19. Pleased	.60	.51	.44	-.04	.42	.58	.32	.60	-.02	.04	.02	.002	.53	-.06	.50	.08	-.01	.04	--

Note: Correlations are all based on at least 124 participants. Correlations .18 and above are significant at $p \leq .05$. Correlations .22 and above are significant at $p \leq .01$. Correlations .29 and above are significant at $p \leq .001$.

Table G

Cluster Centroids for Study 2

	All Participants		
Item	*M* (*SD*) across conditions	Cluster 1 (*n* = 64)	Cluster 2 (*n* = 60)
Grateful	5.23 (1.65)	6	4
Indebted	3.73 (1.98)	5	2
Guilty	1.96 (1.48)	2	2
Moved	3.88 (1.89)	5	2
Uplifted	4.26 (1.77)	5	3
Connected	4.60 (1.83)	5.03	4.10

Note: Centroids are the average of the scores for specific element of the cluster (e.g., Grateful or Moved) for that cluster (e.g., for Cluster 1).

Table H

Table of Counts by Condition and Cluster Assignment for Study 2

Cluster Assignment	Gratitude Experience	Near-Miss Relief	Task-Completion Relief	Total
Cluster 1	27 (62.79%)	25 (49.02%)	12 (40.00%)	64
Cluster 2	16 (37.21%)	26 (50.98)	18 (60.00%)	60
Total	43	51	30	124

Note: Percentages represent proportion of each condition in each cluster. Participants per condition are lower than in other analyses because six participants were listwise deleted for failing to answer at least one of the six variables.

Table I

Effects of Sex and Condition on Dependent Variables in Study 2

Source	*df*	*F*	*p*	Condition	*M(SE)*	
Grateful					Men	Women
Condition	2	4.09	.02	Gratitude Experience:	5.64 (0.28)	5.80 (0.50)
Sex	1	3.34	.07	Near-Miss Relief:	5.37 (0.27)	3.94 (0.40)
Condition X Sex	2	2.47	.09	Task-Completion Relief:	5.29 (0.38)	4.89 (0.37)
Error	123					
Relieved					Men	Women
Condition	2	1.72	.18	Gratitude Experience:	4.49 (0.32)	3.70 (0.58)
Sex	1	6.36	.01	Near-Miss Relief:	5.09 (0.31)	4.06 (0.46)
Condition X Sex	2	0.04	.96	Task-Completion Relief:	5.35 (0.44)	4.50 (0.43)
Error	122					
Indebted					Men	Women
Condition	2	10.38	< .001	Gratitude Experience:	4.88 (0.31)	4.50 (0.56)
Sex	1	5.96	.02	Near-Miss Relief:	3.71 (0.30)	2.13 (0.44)
Condition X Sex	2	1.31	.27	Task-Completion Relief:	3.33 (0.46)	2.77 (0.43)
Error	120					
Guilty					Men	Women
Condition	2	1.05	.35	Gratitude Experience:	2.03 (0.26)	1.00 (0.46)
Sex	1	7.63	.007	Near-Miss Relief:	2.40 (0.25)	1.56 (0.36)
Condition X Sex	2	0.32	.73	Task-Completion Relief:	2.18 (0.35)	1.71 (0.35)
Error	121					
Moved					Men	Women
Condition	2	4.11	.02	Gratitude Experience:	4.39 (0.32)	4.70 (0.58)
Sex	1	2.25	.14	Near-Miss Relief:	4.14 (0.31)	3.06 (0.46)
Condition X Sex	2	1.38	.26	Task-Completion Relief:	3.71 (0.44)	2.89 (0.43)
Error	123					
Uplifted					Men	Women
Condition	2	2.37	.10	Gratitude Experience:	4.76 (0.30)	4.60 (0.55)
Sex	1	3.72	.06	Near-Miss Relief:	4.40 (0.29)	3.19 (.44)
Condition X Sex	2	0.87	.42	Task-Completion Relief:	4.35 (0.42)	3.78 (0.41)
Error	123					
Connected to others					Men	Women
Condition	2	7.67	.001	Gratitude Experience:	5.18 (0.31)	6.10 (0.56)
Sex	1	0.17	.68	Near-Miss Relief:	4.09 (0.30)	4.00 (0.44)
Condition X Sex	2	1.24	.29	Task-Completion Relief:	4.66 (0.44)	4.25 (0.42)
Error	122					
Happy					Men	Women
Condition	2	1.68	.19	Gratitude Experience:	5.18 (0.29)	5.60 (0.52)
Sex	1	1.40	.24	Near-Miss Relief:	5.31 (0.28)	4.06 (0.41)
Condition X Sex	2	2.43	.09	Task-Completion Relief:	5.18 (0.40)	4.89 (0.39)
Error	123					
Worried/anxious					Men	Women
Condition	2	1.45	.24	Gratitude Experience:	2.33 (0.29)	1.60 (0.52)
Sex	1	2.52	.12	Near-Miss Relief:	2.68 (0.28)	2.50 (0.41)
Condition X Sex	2	0.30	.74	Task-Completion Relief:	2.82 (0.40)	2.22 (0.39)
Error	122					
Angry					Men	Women
Condition	2	0.91	.41	Gratitude Experience:	1.88 (0.25)	1.20 (0.45)
Sex	1	6.29	.01	Near-Miss Relief:	2.18 (0.24)	1.75 (0.36)
Condition X Sex	2	0.35	.71	Task-Completion Relief:	2.41 (0.35)	1.44 (0.34)
Error	122					

Table I continued

Source	*df*	*F*	*p*	Condition	*M(SE)*	
Frustrated					Men	Women
Condition	2	1.41	.25	Gratitude Experience:	1.94 (0.26)	1.20 (0.46)
Sex	1	6.35	.01	Near-Miss Relief:	2.47 (0.25)	1.81 (0.37)
Condition X Sex	2	0.01	.99	Task-Completion Relief:	2.38 (0.37)	1.61 (0.35)
Error	120					
Depressed/blue					Men	Women
Condition	2	1.53	.22	Gratitude Experience:	2.03 (0.28)	1.20 (0.52)
Sex	1	3.48	.06	Near-Miss Relief:	2.46 (0.28)	1.75 (0.41)
Condition X Sex	2	0.55	.58	Task-Completion Relief:	2.29 (0.40)	2.17 (0.39)
Error	123					
Joyful					Men	Women
Condition	2	4.94	.009	Gratitude Experience:	4.64 (0.33)	5.40 (0.59)
Sex	1	3.39	.07	Near-Miss Relief:	4.43 (0.32)	2.88 (0.47)
Condition X Sex	2	3.79	.03	Task-Completion Relief:	4.65 (0.45)	3.44 (0.44)
Error	123					
Nervous					Men	Women
Condition	2	1.40	.25	Gratitude Experience:	2.00 (0.27)	1.20 (0.49)
Sex	1	5.67	.02	Near-Miss Relief:	2.46 (0.26)	1.80 (0.40)
Condition X Sex	2	0.02	.98	Task-Completion Relief:	2.53 (0.38)	1.83 (0.37)
Error	122					
Inspired					Men	Women
Condition	2	3.69	.03	Gratitude Experience:	4.46 (0.32)	5.20 (0.59)
Sex	1	3.78	.05	Near-Miss Relief:	4.29 (0.31)	3.00 (0.47)
Condition X Sex	2	3.75	.03	Task-Completion Relief:	5.00 (0.45)	3.44 (0.44)
Error	123					
Scared					Men	Women
Condition	2	1.56	.21	Gratitude Experience:	1.88 (0.26)	1.10 (0.48)
Sex	1	9.98	.002	Near-Miss Relief:	2.46 (0.26)	1.63 (0.38)
Condition X Sex	2	0.15	.86	Task-Completion Relief:	2.65 (0.37)	1.50 (0.36)
Error	123					
Upset					Men	Women
Condition	2	1.91	.15	Gratitude Experience:	1.76 (0.27)	1.00 (0.48)
Sex	1	5.85	.02	Near-Miss Relief:	2.49 (0.26)	1.60 (0.40)
Condition X Sex	2	0.14	.87	Task-Completion Relief:	2.24 (0.37)	1.72 (0.36)
Error	122					
Unhappy					Men	Women
Condition	2	0.59	.56	Gratitude Experience:	1.94 (0.23)	1.10 (0.43)
Sex	1	5.60	.02	Near-Miss Relief:	2.03 (0.23)	1.69 (0.34)
Condition X Sex	2	0.33	.72	Task-Completion Relief:	2.00 (0.33)	1.33 (0.32)
Error	123					
Pleased					Men	Women
Condition	2	4.16	.02	Gratitude Experience:	4.58 (0.29)	5.30 (0.52)
Sex	1	4.31	.04	Near-Miss Relief:	4.77 (0.28)	2.94 (0.41)
Condition X Sex	2	5.43	.006	Task-Completion Relief:	5.00 (0.41)	4.11 (0.39)
Error	122					
Positive Emotion Composite					Men	Women
Condition	2	4.78	.01	Gratitude Experience:	4.81 (0.22)	5.16 (0.40)
Sex	1	4.66	.03	Near-Miss Relief:	4.65 (0.22)	3.46 (0.32)
Condition X Sex	2	3.33	.04	Task-Completion Relief:	4.77 (0.31)	4.02 (0.30)
Error	123					

Table I continued

Source	*df*	*F*	*p*	Condition	*M(SE)*	
Negative Emotion Composite					Men	Women
Condition	2	0.60	.55	Gratitude Experience:	2.28 (0.21)	1.50 (0.38)
Sex	1	8.91	.003	Near-Miss Relief:	2.54 (0.21)	1.84 (0.30)
Condition X Sex	2	0.03	.97	Task-Completion Relief:	2.46 (0.30)	1.82 (0.29)
Error	123					
Emodiversity: All emotions					Men	Women
Condition	2	0.74	.48	Gratitude Experience:	2.49 (0.07)	2.29 (0.13)
Sex	1	5.69	.02	Near-Miss Relief:	2.37 (0.07)	2.22 (0.11)
Condition X Sex	2	0.16	.85	Task-Completion Relief:	2.54 (0.11)	2.28 (0.10)
Error	109					
Emodiversity: Social					Men	Women
emotions	2	2.24	.11	Gratitude Experience:	1.57 (0.07)	1.52 (0.12)
Condition	1	0.89	.35	Near-Miss Relief:	1.43 (0.06)	1.33 (0.09)
Sex	2	0.04	.96	Task-Completion Relief:	1.41 (0.10)	1.34 (0.09)
Condition X Sex	117					
Error						

Note: We found a significant condition by sex interaction on joyful, inspired, pleased, and on the positive emotion composite such that women in the gratitude condition reported more positivity than men in the gratitude condition or men or women in any other condition. We also found a marginal condition by sex interaction on happy and grateful in the same direction. In contrast, past research has found that men benefit more from gratitude exercises than women (Watkins, Uhder, & Pichinevskiy, 2014).

Table J

Means (SDs) and Contrast Tests for Study 3: U.S. Sample

	Experimental Conditions								
	**Gratitude Condition** *Contrast 1: +1* *Contrast 2: +1* *Contrast 3:* 0		**Kindness Condition** *Contrast 1: -1* *Contrast 2: 0* *Contrast 3:* 1		**Control Condition** *Contrast 1: 0* *Contrast 2: -1* *Contrast 3: -1*		*Contrast 2*	*Contrast 3*	*Contrast 4*
	M(SD)	*n*	*M(SD)*	*n*	*M(SD)*	*n*	*t*-contrast or *F*-statistic	*t*-contrast	*t*-contrast
**Grateful (**single-item)	6.45 (1.04)	66	5.53 (1.51)	64	4.06 (1.66)	63	4.05***	9.77***	5.22***
**Gratitude** (multi-item)	5.70 (0.97)	66	5.85 (0.99)	64	5.66 (1.04)	64	-0.85	0.27	1.11
**Indebted**	5.05 (1.84)	66	3.33 (1.93)	64	2.44 (1.57)	64	5.48***	8.33***	2.82**
**Guilty**	2.56 (1.76)	66	2.33 (1.79)	64	2.25 (1.55)	63	0.78	1.02	0.25
**Moved**	5.48 (1.44)	66	4.66 (1.46)	64	2.58 (1.55)	64	3.18**	11.17***	7.92***
**Uplifted**	5.06 (1.71)	66	4.53 (1.44)	64	2.77 (1.56)	64	1.92†	8.31***	6.35***
**Embarrassed**	2.73 (1.30)	66	2.78 (1.44)	64	3.02 (1.59)	64	-0.21	-1.14	-0.92
**Ashamed**	3.34 (1.56)	66	3.79 (1.41)	64	3.77 (1.65)	64	-1.72†	-1.51	0.09
Connectedness (multi-item)	3.72 (0.69)	66	3.57 (0.62)	64	3.58 (0.73)	63	0.94		
Happy	5.85 (1.46)	65	5.13 (1.52)	63	4.11 (1.51)	62	21.49***		
Worried/anxious	2.12 (1.82)	66	1.88 (1.80)	64	2.70 (1.79)	64	3.56*		
Angry	1.62 (1.15)	66	1.44 (1.07)	64	1.70 (1.16)	64	0.93		
Frustrated	2.29 (1.80)	66	2.06 (1.45)	64	2.70 (1.69)	64	2.48†		
Depressed/blue	2.36 (1.55)	66	2.23 (1.65)	64	2.16 (1.63)	64	0.28		
Joyful	3.97 (1.71)	66	4.05 (1.56)	64	3.14 (1.68)	64	5.95**		
Unhappy	1.95 (1.40)	66	2.20 (1.62)	64	2.14 (1.53)	64	0.48		
Pleased	4.45 (1.66)	65	4.45 (1.44)	64	3.69 (1.51)	64	5.25**		
Enjoyment/fun	3.58 (1.91)	64	3.44 (1.60)	64	3.05 (1.63)	64	1.65		
Positive Emotion Composite	4.97 (1.15)	66	4.54 (1.07)	64	3.34 (1.16)	64	36.57***		

Negative Emotion Composite	2.78 (0.96)	66	2.56 (0.95)	64	2.65 (1.08)	64	0.80		
Emodiversity: All emotions	2.36 (0.26)	64	2.35 (0.23)	63	2.25 (0.38)	61	1.70		
**Emodiversity: Social emotions**	1.68 (0.23)	66	1.65 (0.26)	64	1.42 (0.47)	62	0.80	3.94***	3.32***

Note: A *t*-contrast is provided for emotions on which we performed a focused test (emboldened in the table). An omnibus *F*-statistic is reported for all other emotions. The degrees of freedom for the emodiversity analyses are fewer than for the composites because the composites allow for some missing items (we allowed three [out of seven] missing item in the positive emotion composite and up to four [out of nine] missing items in the negative emotion composite), whereas the emodiversity equation does not tolerate any missingness.
†$p \leq .10$. *$p \leq .05$. **$p \leq .01$. ***$p \leq .001$.

Table K

Means (SDs) and Contrast Tests for Study 3: South Korean Sample

	Experimental Conditions								
	**Gratitude Condition** *Contrast 1: +1* *Contrast 2: +1* *Contrast 3:* 0		**Kindness Condition** *Contrast 1: -1* *Contrast 2: 0* *Contrast 3:* 1		**Control Condition** *Contrast 1: 0* *Contrast 2: -1* *Contrast 3: -1*		*Contrast 2*	*Contrast 3*	*Contrast 4*
	M(SD)	*n*	*M(SD)*	*n*	*M(SD)*	*n*	*t*-contrast or *F*-statistic	*t*-contrast	*t*-contrast
**Grateful (**single-item)	6.29 (0.66)	82	4.79 (1.58)	72	4.57 (1.63)	79	7.50***	8.73***	0.85
**Gratitude** (multi-item)	5.47 (1.02)	82	5.39 (1.02)	72	5.17 (1.01)	79	0.48	1.87†	1.33
**Indebted**	4.26 (1.85)	82	2.31 (1.52)	72	2.42 (1.63)	79	7.20***	6.71***	-0.44
**Guilty**	2.65 (1.80)	82	2.03 (1.33)	72	2.94 (1.93)	79	2.44*	-0.99	-3.39***
**Moved**	5.65 (1.02)	82	4.49 (1.39)	72	3.28 (1.65)	79	5.82***	10.91***	4.88***
**Uplifted**	5.23 (1.35)	82	4.65 (1.45)	72	3.76 (1.53)	79	2.48**	6.47***	3.80***
**Embarrassed**	2.08 (1.32)	82	2.06 (1.27)	72	2.56 (1.63)	79	0.08	-2.04*	-2.08*
**Ashamed**	2.61 (1.52)	82	2.69 (1.40)	72	2.33 (1.34)	79	-0.34	1.25	1.54
Connectedness (multi-item)	3.95 (0.54)	82	3.87 (0.63)	72	3.80 (0.66)	79	1.28		
Happy	5.67 (1.17)	82	5.06 (1.58)	72	4.29 (1.63)	79	19.20***		
Worried/anxious	2.79 (1.62)	82	2.94 (1.61)	72	4.30 (1.58)	79	21.28***		
Angry	1.63 (1.09)	82	1.57 (0.92)	72	2.08 (1.31)	79	4.67**		
Frustrated	1.83 (1.23)	82	1.85 (1.29)	72	2.73 (1.76)	79	8.12***		
Depressed/blue	2.15 (1.33)	82	2.08 (1.37)	72	2.99 (1.71)	79	7.77***		
Joyful	4.32 (1.40)	82	4.07 (1.47)	72	3.87 (1.32)	79	2.04		
Unhappy	1.95 (1.44)	82	1.72 (1.14)	72	2.43 (1.46)	79	5.60**		
Pleased	4.72 (1.41)	82	4.31 (1.38)	72	3.96 (1.39)	79	5.97**		
Enjoyment/fun	3.80 (1.54)	82	3.58 (1.50)	72	3.56 (1.38)	79	0.69		
Positive Emotion Composite	5.10 (0.87)	82	4.42 (1.23)	72	3.90 (1.17)	79	24.37***		

Negative Emotion Composite	2.44 (0.97)	82	2.14 (0.90)	72	2.75 (1.12)	79	7.06***		
Emodiversity: All emotions	2.36 (0.22)	82	2.29 (0.33)	72	2.39 (0.25)	79	2.68†		
**Emodiversity: Social emotions**	1.62 (0.22)	82	1.52 (0.33)	72	1.47 (0.38)	79	2.32*	3.09**	0.77

Note: A *t*-contrast is provided for emotions on which we performed a focused test (emboldened in the table). An omnibus *F*-statistic is reported for all other emotions.
†$p \leq .10$. *$p \leq .05$. **$p \leq .01$. ***$p \leq .001$.

Table L

Bivariate Correlations Among Variables in Study 3: Both Cultures

Variable	1	2	3	4	5	6	7	8	9	10	11	12	13	14	15	16	17	18
1. Grateful (single-item)	--																	
2. Gratitude (multi-item)	.35	--																
3. Indebted	.41	-.02	--															
4. Guilty	.10	-.14	.37	--														
5. Moved	.69	.22	.38	.08	--													
6. Uplifted	.60	.21	.27	.02	.75	--												
7. Embarrassed	-.09	-.26	.13	.28	-.11	-.15	--											
8. Ashamed	-.06	-.11	.18	.19	-.04	-.09	.58	--										
9. Connectedness (multi-item)	.21	.50	-.05	-.11	.19	.18	-.41	-.39	--									
10. Happy	.68	.40	.20	-.14	.59	.62	-.22	-.14	.33	--								
11. Worried/anxious	-.12	-.21	.14	.34	-.14	-.09	.30	.19	-.24	-.23	--							
12. Angry	-.13	-.31	.09	.27	-.14	-.10	.42	.27	-.29	-.23	.36	--						
13. Frustrated	-.19	-.22	.09	.32	-.19	-.17	.40	.25	-.27	-.30	.53	.65	--					
14. Depressed/blue	-.13	-.32	.20	.33	-.07	-.09	.45	.28	-.35	-.33	.49	.59	.62	--				
15. Joyful	.37	.29	.01	-.12	.37	.44	-.17	-.15	.38	.55	-.21	-.13	-.23	-.28	--			
16. Unhappy	-.10	-.30	.14	.28	-.04	-.09	.39	.29	-.37	-.32	.46	.59	.60	.74	-.25	--		
17. Pleased	.41	.36	.01	-.15	.42	.47	-.20	-.12	.33	.62	-.25	-.18	-.28	-.38	.79	-.32	--	
18. Enjoyment/fun	.31	.19	.03	-.05	.29	.35	-.03	-.01	.26	.46	-.07	.02	-.10	-.13	.74	-.12	.63	--

Note: Correlations are all based on at least 422 participants. Correlations .10 and above are significant at $p \leq .05$. Correlations .12 and above are significant at $p \leq .01$. Correlations .16 and above are significant at $p \leq .001$.

Table M

Bivariate Correlations Among Variables in Study 3: U.S. Sample

Variable	1	2	3	4	5	6	7	8	9	10	11	12	13	14	15	16	17	18
1. Grateful (single-item)	--																	
2. Gratitude (multi-item)	.28	--																
3. Indebted	.52	-.05	--															
4. Guilty	.16	-.12	.38	--														
5. Moved	.69	.14	.53	.22	--													
6. Uplifted	.61	.09	.44	.14	.81	--												
7. Embarrassed	-.05	-.29	.03	.20	-.01	-.01	--											
8. Ashamed	-.09	-.20	-.003	.10	-.03	-.04	.61	--										
9. Connectedness (multi-item)	.16	.55	-.002	-.12	.08	.06	-.39	-.47	--									
10. Happy	.66	.30	.29	-.10	.55	.61	-.06	-.14	.32	--								
11. Worried/anxious	-.01	-.09	.15	.31	-.01	-.003	.17	.21	-.23	-.11	--							
12. Angry	-.03	-.24	.10	.26	.01	.03	.41	.26	-.31	-.17	.27	--						
13. Frustrated	-.07	-.09	.02	.23	-.06	-.01	.24	.13	-.21	-.13	.44	.68	--					
14. Depressed/blue	.04	-.25	.21	.38	.17	.13	.39	.36	-.41	-.21	.35	.60	.54	--				
15. Joyful	.38	.21	.09	-.09	.35	.42	-.06	-.18	.37	.54	-.17	-.06	-.14	-.23	--			
16. Unhappy	-.02	-.20	.13	.35	.05	-.01	.37	.38	-.40	-.31	.42	.66	.55	.79	-.25	--		
17. Pleased	.33	.25	.07	-.10	.34	.39	-.01	-.12	.31	.57	-.17	-.14	-.17	-.27	.78	-.33	--	
18. Enjoyment/fun	.28	.11	.08	-.02	.25	.33	.06	-.07	.28	.48	-.06	.02	.07	-.12	.76	-.17	.61	--

Note: Correlations are all based on at least 189 participants. Correlations .14 and above are significant at $p \leq .05$. Correlations .18 and above are significant at $p \leq .01$. Correlations .23 and above are significant at $p \leq .001$.

Table N

Bivariate Correlations Among Variables in Study 3: South Korean Sample

**Variable**	**1**	**2**	**3**	**4**	**5**	**6**	**7**	**8**	**9**	**10**	**11**	**12**	**13**	**14**	**15**	**16**	**17**	**18**
1. Grateful (single-item)	--																	
2. Gratitude (multi-item)	.41	--																
3. Indebted	.30	-.05	--															
4. Guilty	.05	-.15	.38	--														
5. Moved	.71	.33	.25	-.06	--													
6. Uplifted	.61	.37	.13	-.11	.68	--												
7. Embarrassed	-.15	.33	.16	.38	-.19	.26	--											
8. Ashamed	-.06	-.17	.27	.32	-.01	-.06	.50	--										
9. Connectedness (multi-item)	.27	.57	-.04	-.13	.28	.27	-.38	-.23	--									
10. Happy	.70	.50	.11	-.18	.63	.65	-.36	-.16	.35	--								
11. Worried/anxious	-.21	-.30	.15	.36	-.28	-.19	.43	.21	-.28	-.34	--							
12. Angry	-.22	-.35	.11	.28	-.29	-.25	.48	.37	-.32	-.29	.44	--						
13. Frustrated	-.32	-.36	.14	.42	-.31	-.34	.54	.35	-.31	.46	.63	.64	--					
14. Depressed/blue	-.29	-.38	.20	.29	-.32	-.33	.54	.28	-.33	-.44	.62	.57	.71	--				
15. Joyful	.37	.43	-.04	-.16	.39	.44	-.25	-.04	.36	.56	-.28	-.22	-.31	-.36	--			
16. Unhappy	-.19	-.41	.15	.23	-.13	-.18	.43	.23	-.35	-.33	.50	.54	.65	.69	-.24	--		
17. Pleased	.50	.48	-.04	-.20	.50	.57	-.37	-.10	.36	.68	-.33	-.23	-.39	-.49	.80	-.32	--	
18. Enjoyment/fun	.35	.32	.01	-.08	.32	.36	-.09	.12	.21	.45	-.10	.00	-.12	-.16	.72	-.05	.66	--

Note: Correlations are all based on at least 233 participants. Correlations .13 and above are significant at $p \leq .05$. Correlations .16 and above are significant at $p \leq .01$. Correlations .21 and above are significant at $p \leq .001$.

Table O

Cluster Centroids for Study 3: Both Cultures

Item	All Participants<br>*M* (*SD*) across conditions	Cluster1 (*n* = 175)	Cluster 2 (*n* = 128)	Cluster 3 (*n* = 122)
Grateful	5.30 (1.63)	6	4	6
Indebted	3.30 (2.00)	3	2	5
Guilty	2.48 (1.73)	2	2	4
Moved	4.38 (1.80)	5	2	5
Uplifted	4.36 (1.71)	5	3	5
Embarrassed	2.51 (1.47)	1.93	2.69	3.15
Ashamed	3.03 (1.58)	2.29	3.12	3.99

Note: Centroids are the average of the scores for specific element of the cluster (e.g., Grateful or Moved) for that cluster (e.g., for Cluster 1).

Table P

Table of Counts by Condition and Cluster Assignment for Study 3: Both Cultures

Cluster Assignment	Gratitude Condition	Kindness Condition	Control Condition	Total
Cluster 1	74 (50.00%)	72 (52.94%)	29 (20.57%)	175
Cluster 2	8 (5.41%)	32 (23.53%)	88 (62.41%)	128
Cluster 3	66 (44.59%)	32 (23.53%)	24 (17.02%)	122
Total	148	136	141	425

Note: Percentages represent proportion of each condition in each cluster. Participants per condition are lower than in other analyses because two participants were listwise deleted for failing to answer at least one of the seven variables.

Table Q

Cluster Centroids for Study 3: U.S. Sample

	All Participants		
Item	*M* (*SD*) across conditions	Cluster1 (*n* = 118)	Cluster 2 (*n* = 74)
Grateful	5.37 (1.72)	6	4
Indebted	3.62 (2.08)	5	2
Guilty	2.38 (1.70)	3	2
Moved	4.25 (1.92)	5	2
Uplifted	4.13 (1.85)	5	3
Embarrassed	2.84 (1.44)	2.73	2.84
Ashamed	3.63 (1.55)	3.54	3.76

Note: Centroids are the average of the scores for specific element of the cluster (e.g., Grateful or Moved) for that cluster (e.g., for Cluster 1).

Table R

Table of Counts by Condition and Cluster Assignment for Study 3: U.S. Sample

Cluster Assignment	Gratitude Condition	Kindness Condition	Control Condition	Total
Cluster 1	61 (92.42%)	46 (71.88%)	11 (17.74%)	118
Cluster 2	5 (7.58%)	18 (28.13%)	51 (82.26%)	74
Total	66	64	62	192

Note: Percentages represent proportion of each condition in each cluster. Participants per condition are lower than in other analyses because two participants were listwise deleted for failing to answer at least one of the seven variables. Participants from the three conditions were not evenly distributed across clusters, $\chi^2(2) = 79.67$, $p <.001$.

Table S

Cluster Centroids for Study 3: South Korean Sample

	All Participants			
Item	*M* (*SD*) across conditions	Cluster1 (*n* = 116)	Cluster 2 (*n* = 68)	Cluster 3 (*n* = 122)
Grateful	5.24 (1.56)	6	4	5
Indebted	3.03 (1.90)	3	2	5
Guilty	2.55 (1.75)	2	2	5
Moved	4.48 (1.69)	6	3	4
Uplifted	4.55 (1.56)	6	3	4
Embarrassed	2.24 (1.43)	1.70	2.21	3.54
Ashamed	2.54 (1.43)	2.09	2.28	3.97

Note: Centroids are the average of the scores for specific element of the cluster (e.g., Grateful or Moved) for that cluster (e.g., for Cluster 1).

Table T

Table of Counts by Condition and Cluster Assignment for Study 3: South Korean Sample

Cluster Assignment	Gratitude Condition	Kindness Condition	Control Condition	Total
Cluster 1	57 (69.51%)	39 (54.17%)	20 (25.32%)	116
Cluster 2	5 (6.10%)	25 (34.72%)	38 (48.10%)	68
Cluster 3	20 (24.39%)	8 (11.11%)	21 (26.58%)	49
Total	82	72	79	233

Note: Percentages represent proportion of each condition in each cluster. Participants per condition are lower than in other analyses because two participants were listwise deleted for failing to answer at least one of the seven variables. Participants from the three conditions were not evenly distributed across clusters, $\chi^2(4) = 46.57$, $p < .001$.

Table U

Effects of Sex, Culture, and Condition on Dependent Variables in Study 3

Source	*df*	*F*	*p*	Condition	*M(SE)*			
Grateful (single-item)					Men/U.S.	Women/U.S.	Men/S.K.	Women/S.K.
Condition	2	73.98	<.001	Gratitude:	6.17 (0.29)	6.62 (0.21)	6.21 (0.24)	6.35 (0.20)
Sex	1	6.14	.01	Kindness:	5.24 (0.34)	5.64 (0.20)	4.51 (0.23)	5.06 (0.23)
Culture	1	0.18	.68	Control:	3.83 (0.33)	4.11 (0.20)	4.41 (0.23)	4.71 (0.21)
Condition X Sex	2	0.17	.85					
Condition X Culture	2	6.16	.002					
Sex X Culture	1	0.02	.89					
Condition X Sex X	2	0.22	.80					
Culture	416							
Error								
Grateful (multi-item)					Men/U.S.	Women/U.S.	Men/S.K.	Women/S.K.
Condition	2	1.05	.35	Gratitude:	5.41 (0.21)	5.86 (0.16)	5.43 (0.17)	5.49 (0.15)
Sex	1	2.81	.09	Kindness:	5.43 (0.24)	6.00 (0.15)	5.57 (0.17)	5.16 (0.17)
Culture	1	9.90	.002	Control:	5.52 (0.24)	5.70 (0.15)	5.08 (0.16)	5.25 (0.16)
Condition X Sex	2	0.24	.78					
Condition X Culture	2	0.64	.53					
Sex X Culture	1	4.95	.03					
Condition X Sex X	2	1.79	.17					
Culture	417							
Error								
Indebted					Men/U.S.	Women/U.S.	Men/S.K.	Women/S.K.
Condition	2	59.78	<.001	Gratitude:	4.74 (0.36)	5.24 (0.27)	4.03 (0.30)	4.42 (0.25)
Sex	1	0.59	.44	Kindness:	3.77 (0.42)	3.17 (0.25)	2.11 (0.28)	2.50 (0.29)
Culture	1	12.33	<.001	Control:	2.28 (0.41)	2.44 (0.25)	2.43 (0.28)	2.41 (0.27)
Condition X Sex	2	0.84	.43					
Condition X Culture	2	4.03	.02					
Sex X Culture	1	0.42	.52					
Condition X Sex X	2	1.11	.33					
Culture	417							
Error								
Guilty					Men/U.S.	Women/U.S.	Men/S.K.	Women/S.K.
Condition	2	1.85	.16	Gratitude:	2.35 (0.36)	2.69 (0.27)	2.62 (0.29)	2.67 (0.25)
Sex	1	0.02	.89	Kindness:	2.71 (0.42)	2.19 (0.25)	1.81 (0.28)	2.22 (0.29)
Culture	1	0.43	.51	Control:	2.44 (0.40)	2.13 (0.25)	3.00 (0.28)	2.88 (0.27)
Condition X Sex	2	0.48	.62					
Condition X Culture	2	3.06	.05					
Sex X Culture	1	0.62	.43					
Condition X Sex X	2	1.01	.37					
Culture	416							
Error								
Moved					Men/U.S.	Women/U.S.	Men/S.K.	Women/S.K.
Condition	2	110.51	<.001	Gratitude:	4.96 (0.29)	5.79 (0.22)	5.56 (0.24)	5.71 (0.20)
Sex	1	8.52	.004	Kindness:	3.94 (0.34)	4.92 (0.21)	4.51 (0.23)	4.47 (0.24)
Culture	1	5.39	.02	Control:	2.56 (0.33)	2.60 (0.20)	2.97 (0.23)	3.55 (0.22)
Condition X Sex	2	0.15	.86					
Condition X Culture	2	1.53	.22					
Sex X Culture	1	1.81	.18					
Condition X Sex X	2	2.57	.08					
Culture	417							
Error								

Table U continued

Source	*df*	*F*	*p*	Condition	*M(SE)*			
Uplifted					Men/U.S.	Women/U.S.	Men/S.K.	Women/S.K.
Condition	2	51.10	<.001	Gratitude:	4.65 (0.31)	5.29 (0.23)	4.97 (0.26)	5.42 (0.22)
Sex	1	5.21	.02	Kindness:	4.06 (0.36)	4.70 (0.22)	4.84 (0.25)	4.47 (0.25)
Culture	1	10.24	.001	Control:	2.78 (0.35)	2.75 (0.22)	3.35 (0.25)	4.12 (0.23)
Condition X Sex	2	0.57	.56					
Condition X Culture	2	2.48	.09					
Sex X Culture	1	0.19	.66					
Condition X Sex X Culture	2	2.80	.06					
Error	417							
Embarrassed					Men/U.S.	Women/U.S.	Men/S.K.	Women/S.K.
Condition	2	3.84	.02	Gratitude:	2.70 (0.30)	2.75 (0.22)	2.02 (0.25)	2.13 (0.21)
Sex	1	0.15	.70	Kindness:	2.77 (0.35)	2.79 (0.21)	1.81 (0.24)	2.33 (0.24)
Culture	1	18.8	<.001	Control:	3.25 (0.34)	3.00 (0.21)	2.62 (0.24)	2.50 (0.22)
Condition X Sex	2	0.79	.46					
Condition X Culture	2	0.07	.93					
Sex X Culture	1	0.60	.44					
Condition X Sex X Culture	2	0.21	.81					
Error	417							
Ashamed					Men/U.S.	Women/U.S.	Men/S.K.	Women/S.K.
Condition	2	1.31	.27	Gratitude:	2.83 (0.31)	3.57 (0.23)	2.37 (0.25)	2.78 (0.21)
Sex	1	5.08	.03	Kindness:	3.38 (0.36)	3.94 (0.21)	2.66 (0.24)	2.75 (0.25)
Culture	1	40.77	<.001	Control:	3.33 (0.35)	3.97 (0.21)	2.54 (0.24)	2.14 (0.23)
Condition X Sex	2	0.81	.45					
Condition X Culture	2	1.78	.17					
Sex X Culture	1	4.10	.04					
Condition X Sex X Culture	2	0.52	.60					
Error	417							
Connectedness (multi-item)					Men/U.S.	Women/U.S.	Men/S.K.	Women/S.K.
Condition	2	1.74	.18	Gratitude:	3.74 (0.13)	3.74 (0.10)	3.97 (0.11)	3.93 (0.09)
Sex	1	1.33	.25	Kindness:	3.60 (0.16)	3.56 (0.09)	3.91 (0.11)	3.81 (0.11)
Culture	1	11.19	.001	Control:	3.74 (0.15)	3.52 (0.09)	3.82 (0.11)	3.77 (0.10)
Condition X Sex	2	0.28	.76					
Condition X Culture	2	0.25	.78					
Sex X Culture	1	0.02	.89					
Condition X Sex X Culture	2	0.31	.74					
Error	417							
Happy					Men/U.S.	Women/U.S.	Men/S.K.	Women/S.K.
Condition	2	34.44	<.001	Gratitude:	5.74 (0.31)	5.93 (0.23)	5.62 (0.26)	5.71 (0.22)
Sex	1	0.82	.37	Kindness:	4.94 (0.36)	5.20 (0.22)	5.08 (0.25)	5.03 (0.25)
Culture	1	0.01	.92	Control:	4.24 (0.36)	4.04 (0.22)	4.00 (0.25)	4.55 (0.23)
Condition X Sex	2	0.02	.98					
Condition X Culture	2	0.34	.71					
Sex X Culture	1	0.13	.72					
Condition X Sex X Culture	2	1.06	.35					
Error	413							

Table U continued

Source	*df*	*F*	*p*	Condition	*M(SE)*			
Worried/anxious					Men/U.S.	Women/U.S.	Men/S.K.	Women/S.K.
Condition	2	17.85	<.001	Gratitude:	2.61 (0.35)	3.33 (0.26)	2.35 (0.29)	3.10 (0.24)
Sex	1	0.66	.42	Kindness:	3.29 (0.41)	2.72 (0.25)	2.95 (0.28)	3.00 (0.28)
Culture	1	0.41	.52	Control:	3.61 (0.40)	2.79 (0.24)	4.46 (0.28)	4.17 (0.26)
Condition X Sex	2	3.17	.04					
Condition X Culture	2	2.24	.11					
Sex X Culture	1	0.03	.86					
Condition X Sex X Culture	2	0.81	.45					
Error	417							
Angry					Men/U.S.	Women/U.S.	Men/S.K.	Women/S.K.
Condition	2	3.89	.02	Gratitude:	1.70 (0.24)	1.55 (0.17)	1.68 (0.19)	1.60 (0.17)
Sex	1	0.33	.57	Kindness:	1.53 (0.27)	1.40 (0.17)	1.43 (0.19)	1.72 (0.19)
Culture	1	1.99	.16	Control:	1.67 (0.27)	1.79 (0.16)	2.32 (0.19)	1.86 (0.17)
Condition X Sex	2	0.42	.66					
Condition X Culture	2	0.80	.45					
Sex X Culture	1	0.02	.88					
Condition X Sex X Culture	2	1.59	.21					
Error	417							
Frustrated					Men/U.S.	Women/U.S.	Men/S.K.	Women/S.K.
Condition	2	9.19	<.001	Gratitude:	1.78 (0.32)	2.48 (0.24)	1.79 (0.27)	1.85 (0.22)
Sex	1	0.52	.47	Kindness:	2.24 (0.38)	2.00 (0.23)	1.81 (0.25)	1.97 (0.26)
Culture	1	0.95	.33	Control:	2.50 (0.36)	2.85 (0.22)	2.92 (0.25)	2.57 (0.24)
Condition X Sex	2	0.71	.49					
Condition X Culture	2	0.52	.60					
Sex X Culture	1	0.97	.33					
Condition X Sex X Culture	2	1.21	.30					
Error	417							
Depressed/blue					Men/U.S.	Women/U.S.	Men/S.K.	Women/S.K.
Condition	2	2.77	.06	Gratitude:	2.13 (0.33)	2.45 (0.24)	2.12 (0.27)	2.17 (0.23)
Sex	1	0.36	.55	Kindness:	2.24 (0.38)	2.23 (0.23)	1.95 (0.26)	2.31 (0.26)
Culture	1	1.25	.27	Control:	2.11 (0.37)	2.29 (0.23)	3.16 (0.26)	2.83 (0.24)
Condition X Sex	2	0.28	.75					
Condition X Culture	2	3.70	.03					
Sex X Culture	1	0.19	.66					
Condition X Sex X Culture	2	0.63	.53					
Error	417							
Joyful					Men/U.S.	Women/U.S.	Men/S.K.	Women/S.K.
Condition	2	5.49	.004	Gratitude:	4.26 (0.32)	3.86 (0.23)	4.47 (0.26)	4.21 (0.22)
Sex	1	3.64	.06	Kindness:	3.94 (0.37)	4.09 (0.22)	4.32 (0.25)	3.81 (0.25)
Culture	1	3.53	.06	Control:	3.78 (0.36)	2.88 (0.22)	3.78 (0.25)	3.95 (0.23)
Condition X Sex	2	0.12	.89					
Condition X Culture	2	0.81	.45					
Sex X Culture	1	0.35	.56					
Condition X Sex X Culture	2	2.53	.08					
Error	417							

Table U continued

Source	*df*	*F*	*p*	Condition	*M(SE)*			
Unhappy					Men/U.S.	Women/U.S.	Men/S.K.	Women/S.K.
Condition	2	2.50	.08	Gratitude:	1.61 (0.30)	2.05 (0.22)	2.06 (0.25)	1.88 (0.21)
Sex	1	0.08	.78	Kindness:	2.53 (0.35)	2.09 (0.21)	1.76 (0.24)	1.78 (0.24)
Culture	1	0.06	.81	Control:	2.06 (0.34)	2.23 (0.21)	2.57 (0.24)	2.31 (0.22)
Condition X Sex	2	0.44	.64					
Condition X Culture	2	2.92	.06					
Sex X Culture	1	0.44	.51					
Condition X Sex X Culture	2	1.27	.28					
Error	417							
Pleased					Men/U.S.	Women/U.S.	Men/S.K.	Women/S.K.
Condition	2	8.57	<.001	Gratitude:	4.64 (0.31)	4.38 (0.23)	4.91 (0.25)	4.58 (0.21)
Sex	1	0.58	.45	Kindness:	4.18 (0.35)	4.55 (0.21)	4.38 (0.24)	4.22 (0.24)
Culture	1	0.48	.49	Control:	4.17 (0.34)	3.46 (0.21)	3.76 (0.24)	4.14 (0.23)
Condition X Sex	2	0.61	.54					
Condition X Culture	2	0.35	.71					
Sex X Culture	1	0.29	.59					
Condition X Sex X Culture	2	2.55	.08					
Error	416							
Enjoyment/fun					Men/U.S.	Women/U.S.	Men/S.K.	Women/S.K.
Condition	2	1.79	.17	Gratitude:	3.57 (0.33)	3.62 (0.25)	4.21 (0.27)	3.52 (0.23)
Sex	1	1.61	.21	Kindness:	3.29 (0.38)	3.49 (0.23)	3.76 (0.26)	3.42 (0.26)
Culture	1	2.96	.09	Control:	3.50 (0.37)	3.85 (0.23)	3.46 (0.26)	3.64 (0.25)
Condition X Sex	2	0.19	.83					
Condition X Culture	2	0.10	.91					
Sex X Culture	1	0.21	.65					
Condition X Sex X Culture	2	2.31	.10					
Error	417							
Positive Emotion Composite					Men/U.S.	Women/U.S.	Men/S.K.	Women/S.K.
Condition	2	51.48	<.001	Gratitude:	4.86 (0.23)	5.06 (0.17)	5.13 (0.19)	5.06 (0.16)
Sex	1	0.60	.44	Kindness:	4.23 (0.27)	4.66 (0.16)	4.49 (0.18)	4.35 (0.19)
Culture	1	3.24	.07	Control:	3.55 (0.26)	3.23 (0.16)	3.68 (0.18)	4.10 (0.17)
Condition X Sex	2	0.07	.94					
Condition X Culture	2	1.73	.18					
Sex X Culture	1	0.02	.90					
Condition X Sex X Culture	2	2.92	.06					
Error	417							
Negative Emotion Composite					Men/U.S.	Women/U.S.	Men/S.K.	Women/S.K.
Condition	2	3.17	.04	Gratitude:	2.49 (0.21)	2.90 (0.15)	2.34 (0.17)	2.51 (0.14)
Sex	1	0.66	.42	Kindness:	2.72 (0.24)	2.50 (0.15)	2.03 (0.16)	2.29 (0.17)
Culture	1	3.99	.05	Control:	2.58 (0.24)	2.72 (0.14)	2.89 (0.16)	2.63 (0.15)
Condition X Sex	2	1.12	.33					
Condition X Culture	2	2.51	.08					
Sex X Culture	1	0.07	.79					
Condition X Sex X Culture	2	1.62	.20					
Error	417							

Table U continued

Source	*df*	*F*	*p*	Condition	*M(SE)*			
Emodiversity: All emotions					Men/U.S.	Women/U.S.	Men/S.K.	Women/S.K.
Condition	2	0.24	.79	Gratitude:	2.31 (0.06)	2.38 (0.04)	2.38 (0.05)	2.34 (0.04)
Sex	1	0.11	.74	Kindness:	2.40 (0.07)	2.33 (0.04)	2.27 (0.05)	2.32 (0.05)
Culture	1	0.28	.59	Control:	2.34 (0.07)	2.22 (0.04)	2.36 (0.05)	2.22 (0.04)
Condition X Sex	2	0.26	.77					
Condition X Culture	2	2.83	.06					
Sex X Culture	1	1.20	.27					
Condition X Sex X Culture	2	2.12	.12					
Error	411							
Emodiversity: Social emotions					Men/U.S.	Women/U.S.	Men/S.K.	Women/S.K.
Condition	2	11.96	<.001	Gratitude:	1.65 (0.07)	1.70 (0.05)	1.62 (0.06)	1.62 (0.05)
Sex	1	0.75	.39	Kindness:	1.64 (0.08)	1.65 (0.05)	1.46 (0.05)	1.58 (0.05)
Culture	1	2.11	.15	Control:	1.48 (0.08)	1.39 (0.05)	1.43 (0.05)	1.51 (0.05)
Condition X Sex	2	0.31	.73					
Condition X Culture	2	1.83	.16					
Sex X Culture	1	1.14	.29					
Condition X Sex X Culture	2	1.00	.37					
Error	415							

Note: The Factorial ANOVA results of the condition by culture interaction reported in the main manuscript are from analyses run on just condition and culture effects, without sex in the model. The results presented in Table 25 should be taken with caution as our U.S. sample was 70.5% female (S.K. sample was 54.08% female), and therefore we had fewer male participants from which to draw inferences. No consistent patterns emerged except for a main effect of condition on most of our hypothesized variables (consistent with the results reported in the manuscript).
